# tsCRISPR based identification of Rab proteins required for the recycling of *Drosophila* TRPL ion channel

**DOI:** 10.3389/fcell.2024.1444953

**Published:** 2024-09-20

**Authors:** Matthias Zeger, Lena Sarah Stanisławczyk, Marija Bulić, Andrea Maria Binder, Armin Huber

**Affiliations:** Department of Biochemistry, Institute of Biology, University of Hohenheim, Stuttgart, Germany

**Keywords:** *Drosophila* photoreceptors, protein transport in polarized cells, recycling pathway, TRPL ion channel, Rab proteins, endosomal trafficking, endoplasmic reticulum, trans-Golgi

## Abstract

In polarized cells, the precise regulation of protein transport to and from the plasma membrane is crucial to maintain cellular function. Dysregulation of intracellular protein transport in neurons can lead to neurodegenerative diseases such as Retinitis Pigmentosa, Alzheimer’s and Parkinson’s disease. Here we used the light-dependent transport of the TRPL (transient receptor potential-like) ion channel in *Drosophila* photoreceptor cells to study the role of Rab proteins in TRPL recycling. TRPL is located in the rhabdomeric membrane of dark-adapted flies, but it is transported out of the rhabdomere upon light exposure and localizes at the Endoplasmatic Reticulum within 12 h. Upon subsequent dark adaptation, TRPL is recycled back to the rhabdomeric membrane within 90 min. To screen for Rab proteins involved in TRPL recycling, we established a tissue specific (ts) CRISPR/Cas9-mediated knock-out of individual *Rab* genes in *Drosophila* photoreceptors and assessed TRPL localization using an eGFP tagged TRPL protein in the intact eyes of these mutants. We observed severe TRPL recycling defects in the knockouts of *Rab3*, *Rab4*, *Rab7*, *Rab32*, *and RabX2*. Using immunohistochemistry, we further showed that Rab3 and RabX2 each play a significant role in TRPL recycling and also influence TRPL transport. We localized Rab3 to the late endosome in *Drosophila* photoreceptors and observed disruption of TRPL transport to the ER in *Rab3* knock-out mutants. TRPL transport from the ER to the rhabdomere ensues from the trans-Golgi where RabX2 is located. We observed accumulated TRPL at the trans-Golgi in *RabX2* knock-out mutants. In summary, our study reveals the requirement of specific Rab proteins for different steps of TRPL transport in photoreceptor cells and provides evidence for a unique retrograde recycling pathway of TRPL from the ER via the trans-Golgi.

## 1 Introduction

The regulation of membrane protein trafficking is vital for preserving the integrity and functionality of photoreceptor cells, as it governs the incorporation of receptors and ion channels into the plasma membrane (PM). Intracellular transport of plasma membrane proteins starts after the synthesis of proteins at the rough endoplasmic reticulum (ER) and proceeds through anterograde transport via the Golgi network to reach the plasma membrane (Barlowe and Miller, 2013; Glick and Luini, 2011). Conversely, membrane proteins are eliminated from the plasma membrane through endocytosis, by typically entering early endosomes (Huotari and Helenius, 2011; Maxfield and McGraw, 2004), which subsequently mature into late endosomes (Raiborg and Stenmark, 2009; Wang et al., 2011). In the endosomes, a crucial decision is made regarding the destiny of the endocytosed proteins, as they can either enter the lysosomal pathway for degradation or exit the endosome for recycling (Burd and Cullen, 2014; Cullen and Korswagen, 2012). In the recycling pathway, a protein may be recycled directly from the early endosome, referred to as “the fast recycling pathway,” or indirectly as a slower recycling pathway through a distinct subgroup of recycling endosomes called the endosomal recycling compartment (Grant and Donaldson, 2009).

In the context of protein trafficking, Rab GTPases (Ras-related in brain) play a pivotal role as regulators due to their ability to cycle between an active conformation prompted by GTP (guanosine triphosphate) binding and an inactive conformation upon hydrolysis of GTP to GDP (guanosine diphosphate) (Pfeffer, 2017; Stenmark, 2009; Zerial and McBride, 2001; Zhen and Stenmark, 2015). Activated Rab GTPases recruit a diverse array of effector proteins with various intracellular functions, thus they may influence cargo selection, formation of carrier vesicles from donor membranes, vesicle transportation along cytoskeletal tracks, and tethering and fusion of carrier vesicles with the target membrane (Gillingham et al., 2014; Stenmark, 2009; Gurkan et al., 2005). Notably, certain Rab proteins, including Rab3, Rab4, Rab11, Rab26, Rab25, Rab32 and Rab35 are implicated in protein recycling processes (Chan et al., 2011; Daro et al., 1996; Diaz-Rohrer et al., 2023; Dozynkiewicz et al., 2012; Hamelin et al., 2005; Kouranti et al., 2006; Ullrich et al., 1996; Van Der Sluijs et al., 1992). A defective recycling affects the protein level equilibrium in the cell, potentially causing aggregation of proteins, a characteristic feature observed in various neurodegenerative disorders (Wang et al., 2014). Therefore, it is not surprising that Rab proteins are associated with various neurodegenerative diseases. (Cherry et al., 2013; Dhekne et al., 2018; Kiral et al., 2018; Spinosa et al., 2008; Verhoeven et al., 2003).

The compound eye of *Drosophila melanogaster* has emerged as a model system for investigating the mechanisms involved in membrane protein trafficking in living organisms. In the photoreceptor cells of *Drosophila*, protein trafficking ensures that the appropriate quantity of phototransduction proteins, such as rhodopsin and the light-activated TRP (transient receptor potential) and TRPL (TRP-Like) ion channels, are transported from their site of synthesis in the ER to the light-absorbing apical plasma membrane (Kunduri et al., 2014; Li et al., 2007; Satoh et al., 2005; Satoh et al., 1997; Schopf and Huber, 2017). This membrane region, referred to as the rhabdomere, is characterized by a densely packed arrangement of microvilli along one side of the cell (Hardie, 1985). TRPL, an ion channel of the phototransduction pathway undergoes light-dependent translocation between the rhabdomere (its location in dark-adapted flies) and the ER, where to it is transported within several hours following light stimulation (Wagner et al., 2022a). While a fraction of TRPL is degraded via the endolysosomal pathway, the majority is stored in the cell body and is subsequently recycled (Bähner et al., 2002). Upon cessation of illumination and ensuing transfer to darkness, recycled TRPL is transported back to the rhabdomere within 90 min (Wagner et al., 2022a). Hence the translocation of TRPL provides a great model system for investigating the stimulus-induced/regulated recycling of plasma membrane proteins. In a previous study, we investigated the role of Rab proteins in TRPL internalization using flies that expressed dominant negative Rab variants and found that Rab5 and RabX4 are important for TRPL internalization (Oberegelsbacher et al., 2011). Rab proteins are evolutionary conserved, and of the 28 Rab or Rab-related proteins in *Drosophila*, 23 have direct human orthologs with at least 50% sequence identity (Chan et al., 2011; Jin et al., 2012; Zhang et al., 2007).

Here, we focus on examining the role of Rab proteins in TRPL recycling. To investigate this, we established an eye-specific *CRISPR/Cas9* driver line to generate knock-out mutants of *Drosophila Rab* genes in a tissue-specific manner. We screened these mutants for defects in TRPL recycling using water immersion (WI) microscopy and found that *Rab3, Rab4, Rab7, Rab32, and RabX2* exhibited the most significant defects in recycling. Immunohistochemistry (IHC) analysis of the recycling defects revealed a novel retrograde recycling pathway for TRPL via the trans-Golgi mediated by RabX2. Moreover, we showed that the transport of internalized TRPL from the late endosome to the ER is dependent on Rab3.

## 2 Materials and methods

### 2.1 Fly husbandry and illumination conditions

Flies were reared and maintained on a yeast/cornmeal diet (84 gL^-1^ fresh baker’s yeast, 50 gL^−1^ cornmeal, 32 gL^−1^ sucrose, 12 gL^−1^ agar, supplemented with methylparaben, vitamin C, and propionic acid) at a temperature of 25°C. For dark-adaptation, flies were kept in the dark for the indicated period at 25°C. For illumination in TRPL translocation experiments lasting up to 16 h, flies were exposed to a white fluorescent tube (4000 K, 1750 Lux, E_e_^470 nm^ = 298 μW cm^−2^, E_e_^590 nm^ = 215 μW cm^−2^, Osram, Munich, Germany) at room temperature. Orange colored transparent plastic boxes were used in combination with the fluorescent tube to illuminate flies with orange light (>560 nm, E_e_^590 nm^ = 115 μW cm^−2^). Dark-adapted flies were prepared for subsequent experiments in the dark using only a weak cold light source (KL 1500 LCD, Schott, Mainz, Germany) with a deep red long pass filter (RG630, Schott). Light-adapted flies were prepared in white room light. To inhibit protein biosynthesis in adult photoreceptor cells, flies were treated with 35 mM cyclohexminide (CHX) in apple juice containing 1.5% sucrose for the indicated time period following a 16 h starvation period. The uptake was confirmed by the use of food coloring.

### 2.2 Fly stocks used

Fly lines were obtained from the Bloomington *Drosophila* stock center (BL), Vienna *Drosophila* Resource Center (VDRC), or indicated research groups.

#### 2.2.1 Promoter lines

*Rh1 > Gal4* (BL ID: 8691), *Ey > Gal4* (BL ID: 5534)*, GMR > Gal4* (BL ID: 9146), *Rh1 > Gal4* (Yamashita et al., 2022).

#### 2.2.2 Cas9 lines

*UAS > uXS-Cas9* (VDRC ID: 340000), *UAS > uS-Cas9* (VDRC ID: 340001), *UAS > uM-Cas9* (VDRC ID: 340002).

#### 2.2.3 Driver Cas9 line provided

w*; GMR > Gal4 UAS > uM-Cas9/CyO (Port et al., 2020).

#### 2.2.4 Driver lines generated in this study by standard *Drosophila* genetics

w*; UAS > uS-Cas9/CyO; Rh1 > Gal4/MKRS, w*; UAS > uM-Cas9/CyO; Rh1 > Gal4/MKRS, w*; ey > Gal4 UAS > uXS-Cas9/CyO, w*; ey > Gal4 UAS > uS-Cas9/CyO, *P[GMR-w.IR]13D/FM7; ey > Gal4 UAS > uS-Cas9/CyO*, *P[GMR-w.IR]13D/FM7; ey > Gal4 UAS > uS-Cas9/CyO; TRPL::eGFP/MKRS*.

#### 2.2.5 sgRNA lines

*sgRNA-norpA* (VDRC ID: 341777), *sgRNA-vps35* (BL ID: 76385), *sgRNA-RabX1* (BL ID: 80901), *sgRNA-RabX2* (BL ID: 81748), *sgRNA-RabX4* (BL ID: 81901), *sgRNA-RabX5* (VDRC ID: 342953), *sgRNA-RabX6* (VDRC ID: 342914), *sgRNA-Rab1* (VDRC ID: 342882), *sgRNA-Rab2* (BL ID: 96322), *sgRNA-Rab3* (BL ID: 81906), *sgRNA-Rab4* (VDRC ID: 342599), *sgRNA-Rab5* (VDRC ID: 342598), *sgRNA-Rab6* (VDRC ID: 342913), *sgRNA-Rab7* (VDRC ID: 342597), *sgRNA-Rab8* (BL ID: 81899), *sgRNA-Rab9* (VDRC ID: 342596), *sgRNA-Rab10* (BL ID: 91952), *sgRNA-Rab11* (VDRC ID: 342893), *sgRNA-Rab14* (BL ID: 96336), *sgRNA-Rab19* (VDRC ID: 34237), *sgRNA-Rab21* (VDRC ID: 342623), *sgRNA-Rab23* (BL ID: 76452), *sgRNA-Rab26* (VDRC ID: 342620), *sgRNA-Rab27* (BL ID: 77014), *sgRNA-Rab30* (VDRC ID: 342968), *sgRNA-Rab32* (VDRC ID: 342622), *sgRNA-Rab39* (BL ID: 84008)*, sgRNA-Rab40* (VDRC ID: 342616).

#### 2.2.6 Control lines

w* (BL ID: 5), *w, norpA[P24]* (BL ID: 9048), *Rab3[rup]* (BL ID: 78045)

#### 2.2.7 Myc-tagged lines

*w[1118]; TI{TI}Rab3[EYFP]* (BL ID: 62541), *w[1118]; TI{TI}Rab7[EYFP]* (BL ID: 62545).

#### 2.2.8 YFP-tagged lines under UAS control

y[1] w[*]; P{w[+mC] = UASp-YFP.RabX2}idc[08]/TM3, Sb[1] (BL ID: 23275).

### 2.3 Tissue specific mutagenesis using tsCRSPR/Cas9

tsCRISPR based mutagenesis was performed as described, by crossing driver lines containing *UAS-Cas9* and *Gal4* under control of different tissue specific promoters with flies expressing sgRNAs (Port et al., 2020). For establishing eye-specific tsCRISPR sgRNA directed against *norpA* (VDRC ID: 341777) was used and mutagenesis efficiency was tested by immunoblot analysis and ERG measurements (see results). In our study we focused further on *Rab3* and *RabX2*. Efficient gene disruption by the sgRNA line directed against *Rab3* was shown previously (Allen et al., 2021; Sachidanandan et al., 2023). To verify efficient gene disruption for *RabX2*, we employed T7 endonuclease (NEB, # M0689L) digestion of hybridized PCR amplified genomic regions covering the sgRNA target sites of the *RabX2* gene (ALLin™ Mega HiFi Mastermix (highQu, Cat#HLM0201), forward primer: CGA​CTT​GAC​GAT​GAG​CCA​CTT; reverse primer: CAC​ATG​GCG​CCG​TAT​CTC​CTT). The method relies on the characteristic of T7 endonuclease to digest DNA at base pair mismatches. Mutations induced by tsCRISPR are indicated by appearance of DNA bands smaller than the PCR amplificat. We amplified DNA obtained from single eyes of *RabX2* gene disruption flies and found that 10 out of 10 eyes contained mutations in the amplified DNA.

### 2.4 Immunoblot analysis

For protein extracts from a single head, one fly head was homogenized in 10 µL SDS extraction buffer (4% (w/v) SDS, 1 mM EDTA, 75 mM Tris/HCl, pH 6.8) and incubated for 10 min at room temperature. Extracts were mixed with 0.2 volumes of 5× SDS sample buffer (5% (w/v) SDS, 30% (v/v) glycerol, 5% (v/v) 2-mercaptoethanol, 100 μg mL^-1^ bromophenol blue, 500 mM Tris/HCl, pH 6.8). Samples were boiled at 95°C for 1 min, loaded onto 10% polyacrylamide gels, separated by SDS-PAGE, and transferred to a PVDF-membrane (Bio-Rad, Hercules, CA, United States). Membranes were blocked for 10 min in TBS-T (0.1% (v/v) Tween^®^ 20, 5% (w/v) skim milk, 150 mM NaCl, 10 mM Tris/HCl, pH 7.5) with 5% skim milk. After blocking, membranes were incubated with primary antibody in TBS-T with 5% skim milk overnight at 4°C. Antibodies were used at the following concentrations: rabbit α-NorpA, 1:1,000 (Huber et al., 2000) and mouse α-Tubulin (DSHB Cat# E7, RRID: AB_579793), 1:500. PVDF-membranes were subsequently washed three times with TBS-T for 5 min each and then incubated with secondary antibody in TBS-T with 5% skim milk for 2 h at room temperature. HRP-conjugated secondary antibodies were used 1:10,000 (Sigma-Aldrich # A0545; # A9044). After three final washing steps in TBS-T, 5 min each, signals were detected by enhanced chemiluminescence (0.091 M Tris/HCl pH 8.6; 0.0227% (w/v) luminol; 0.01% (w/v) para-hydroxycoumaric acid; 0.01% H_2_O_2_) with the ChemiDocTM XRS + Molecular Imager^®^ using the software Quantity One 4.6.9 (Bio-Rad). Quantification of immunoblot signals was performed with Image Lab 6.0.1 (Bio-Rad) by integration of the pixel intensities of each protein band. NorpA signals were normalized using α -Tubulin signals of the same sample. At least 3 individual flies were analyzed per data point.

### 2.5 Electroretinography

For ERG recordings, flies were briefly immobilized on ice and fixed inside of improvised yokes made from 200 µL pipette tips before they were mounted in the center of a Faraday cage. Chlorinated silver wires were inserted into glass micropipettes filled with Davenport solution (100 mM NaCl_2_, 2 mM KCl, 1 mM CaCl_2_, 1.8 mM NaHCO_3_, pH 7.2) and utilized as electrodes. Glass micropipettes were pulled from capillaries BF150 75 10 (Sutter Instrument, Tuttlingen, Germany) using a PC-10 puller (Narishige Scientific Instrument Lab., Tokyo, Japan). Silver wires with 250 μm diameter (Good Fellow, Huntingdon, United Kingdom) were chlorinated with an ACl-01 automatic chlorider (NPI Electronics, Tamm, Germany). The recording electrode was inserted into the eye just below the cornea and the reference electrode into the head capsule. Electrodes had a resistance of ca. 30 mΩ when immersed in a 0.9% NaCl solution. Light stimuli lasting 5 s were generated by a PLED-02M (NPI Electronics) driven orange light emitting diode M3A1 HY 30, 590 nm (Roithner Lasertechnik, Vienna, Austria) in a setup of two collimating lenses (Linos, Göttingen, Germany) within the light path. The light intensity was determined to be at 3.58 mW cm^−2^ at the point where the fly eye would be. ERG recordings were performed at room temperature after 3 min of dark-adaptation. An EXT 10-2F amplifier (NPI Electronics) was used with a 700 Hz low pass filter. Data recording was achieved by the whole cell analysis software WinWCP V4.7.6 (University of Strathclyde, Glasgow, Scotland; RRID: SCR_014713). The maximum of the resulting depolarization amplitude was measured from the baseline while omitting on-/off-transients 15–21 individual flies were analyzed per data point.

### 2.6 Water immersion microscopy

Water immersion microscopy of living flies was conducted following the method described by Wagner et al. (2022b). TRPL::eGFP expressing flies were subjected to ice anesthesia for 15–30 min. Subsequently, the flies were carefully placed inside a 200 µL pipette tip and gently pushed towards the tip using compressed air. The pipette tip was then trimmed just in front of the head, and the fly was cautiously repositioned a few millimeters back into the pipette using tweezers. After that, the pipette tip was trimmed again, leaving only the head of the fly protruding from the tip. The pipette, along with the fly, was attached to an object slide using plastine, ensuring that an eye of the fly was facing upwards. Prior to capturing images, a large drop of chilled water was applied to a water immersion objective, and the eye was focused by covering it with the water drop. The eGFP fluorescence in the fly eyes was observed using the Carl Zeiss microscope AxioImager.Z1m (water immersion objective: Achroplan 20x/0.50W Ph2). Images were acquired using the Axiocam 530 mono (Carl Zeiss) camera and the ZEN 2 (blue edition) software (Carl Zeiss). After image acquisition the fly was returned to a vial. The same fly was used for different illumination conditions (d, dl, dld). The exposure time was individually adjusted at the first analyzed time-point, such that it remained just below overexposure. The same exposure time was then applied for each subsequent analysis time point.

### 2.7 Immunostaining of fly eyes and fluorescence microscopy

Sample preparation for conventional fluorescence microscopy was performed as followed: *Drosophila* fly heads were separated from the body and dissected into two halves. The halves were fixed in a solution containing 2% (w/v) paraformaldehyde (PFA) in PBS (137 mM NaCl, 2.7 mM KCl, 10 mM Na_2_HPO_4_, and 2 mM KH_2_PO_4_, pH 7.2) for 45–60 min at room temperature. After fixation, the semi-heads were washed twice with phosphate buffer (77 mM Na_2_HPO_4_ and 23 mM NaH_2_PO_4_, pH 7.4) for 10 min. Subsequently, sucrose infiltration was performed through two washing steps: first in 10% (w/v) sucrose and then in 25% (w/v) sucrose in phosphate buffer, both at room temperature for 45 and 30 min, respectively. The eyes were further infiltrated with 50% (w/v) sucrose in phosphate buffer overnight at 4°C and then embedded in Tissue-Tek^®^ O.C.T. (Sakura, Tokyo, Japan). Cryosections of *Drosophila* eyes were obtained at −25°C using a Cryostr NX50 cryostat (epredia) with a thickness of 10 µm. The sections were briefly fixed in a solution containing 2% (w/v) PFA in PBS for 10 min at room temperature, followed by three washes in PBS for 5 min each. For immunostaining, the sections were blocked in PBS-T (1% (w/v) BSA, 0.3% (v/v) Triton X-100 in PBS) for 2 h at room temperature. After blocking, the sections were incubated overnight at 4°C with primary antibodies diluted in PBS-T. The concentrations of the primary antibodies used were as follows: rabbit α-TRPL (Bähner et al., 2002), 1:20; guinea pig α-TRPL (Richter et al., 2011), 1:10; mouse α-Calnexin99A (DSHB Cat# Cnx99A 6-2-1, RRID: AB2722011), 1:10; goat α-Golgin245 (DSHB Cat# Golgin245, RRID: AB_2569587), 1:2000; rabbit α-Myc (invitrogen, Cat# PA1-981), 1:500; mouse α-Myc (Cell Signaling Technology, Cat# 2276), 1:500 and mouse α-Rab7 (DSHB, Cat# Rab7, RRID: AB_2722471), 1:20. Following primary antibody incubation, the sections were washed three times with PBS for 5 min each. Subsequently, the sections were incubated with secondary antibodies (Thermo Fisher Scientific, Cat# A-31562; A-11008; A-21084; A-10035; A-21450) diluted in PBS-T for 2 h at room temperature. In conventional microscopy, AF 488-, AF 660-, AF 680- and Cy5-conjugated secondary antibodies were used at a dilution of 1:100. Additionally, AF 546 conjugated phalloidin (Thermo Fisher Scientific, Cat# A-22283), 1:600 as well as DAPI (Merck, Darmstadt, Germany; Cat# D9542), 1:10,000 were included in the secondary antibody solution for staining F actin in rhabdomeres or the nuclei of photoreceptor cells, respectively. After three final washes in PBS for 5 min each, the sections were mounted in Mowiol 4–88 with the addition of 2% n-propyl gallate as an anti-fading agent. For analysis of slices by fluorescence microscopy, images were acquired using an AxioImager.Z1m microscope (objective: EC Plan-Neofluar 40×/1.3 Oil) equipped with the ApoTome module (Carl Zeiss, Jena, Germany). The Axiocam 530 mono (Carl Zeiss) camera and ZEN 2 (blue edition) software (Carl Zeiss) were used for image capture.

### 2.8 Quantitative analysis of fluorescence images

Quantitative analysis of relative TRPL fluorescence in the rhabdomeres was performed using ImageJ 2.3.0 software (RRID: SCR_003070). The relative amount of TRPL in the rhabdomeres (R) was calculated using the formula $R=\frac{I_{R}}{I_{B}}$ where *I*_*R*_ and *I*_*B*_ represent the fluorescence intensities in the rhabdomeres R1-6 and R7, respectively. For each eye, three ommatidia were analyzed, and measurements from at least three individual flies were averaged per data point. The value obtained for the respective dark-raised fly was set to 100%.

In order to quantify the colocalization between TRPL and Golgin245, TRPL and Calenxin99A, Rab3-Myc and TRPL, Rab7-Myc and TRPL, Rab3-Myc and Calenxin99A, RabX2-YFP and Golgin245, Pearson’s correlation coefficients (PCC) were determined. Pearson’s correlation coefficient (PCC) analysis of fluorescence micrographs was conducted as previously described (Dunn et al., 2011; Manders et al., 1993; Mcdonald and Dunn, 2013). In brief, all measurements were conducted in a semi-blind manner, with one of the two channels to be tested for colocalization being deactivated. The phalloidin signal was used for orientation purposes. When examining the colocalization of TRPL with Calnexin99A, Rab7-Myc, Golgin245, and Rab3-Myc, the TRPL signal was activated while the signal of the second protein was deactivated. Quantification of the colocalization between Rab3-Myc and Rab7 or Calnexin99A was conducted with the Rab3-Myc signal activated, colocalization between Golgin245 and RabX2-YFP was performed with activated Golgin245 signal. The Zeiss Zen2 software then used pixel intensities of both channels of interest in the semi-blind selected areas to calculate Pearson correlation using the formula: $\mathrm{PCC}=\frac{\sum_{i}\left(R_{i}-\bar{R}\right)∙\left(G_{i}-\bar{G}\right)}{\sqrt{\sum_{i}{\left(R_{i}-\bar{R}\;\right)}^{2}∙{\left(G_{i}-\bar{G}\right)}^{2}}}$ . $R_{i}$ and $G_{i}$ represent the intensity values of the red and green channels, respectively, of pixel *i*, while $\bar{R}$ and $\bar{G}$ represent the mean intensities of the red and green channels (Dunn et al., 2011). The PCC scale ranges from −1 (total anti-correlation) to 1 (total correlation), with a value of zero indicating no correlation of fluorescent signals. To reduce background, intensity thresholds were set to 10% of the maximal signal for Cy5 signals (Cnx99A, Golgin245, Rab7, Rab7-Myc, Rab3-Myc) and to 20% for AF488 signals (TRPL, RabX2-YFP, Rab3-Myc). The cross-sectional area of the photoreceptor cells (PRCs) was measured using a circle of 32 pixels which corresponds approximately to the cell body and rhabdomere of one photoreceptor cell. In longitudinal sections circles of 12.16 pixels were positioned next to the rhabdomere. Per section six ommatidia were evaluated and the average of five measurements per ommatidium were taken as a data point. As indicated in the figure legends 3-6, sections (biological replicates) obtained from different flies were analyzed.

### 2.9 Statistical analysis

Statistical analyses were conducted using GraphPad Prism 9.3.1 (GraphPad Software, San Diego, CA; RRID: SCR_002798). The statistical analysis of the data involved the use of an unpaired t-test (two-tailed) for evaluations between two means. For analyses involving more than two means, a one-way analysis of variance (ANOVA) was conducted. To assess the effects of two independent factors and their interaction, a two-way ANOVA was utilized. Post-hoc comparisons following the ANOVAs were carried out using the Bonferroni correction to adjust for multiple testing. The results of the statistical tests are indicated in the graphs by asterisks above horizontal bars connecting the compared groups. These asterisks denote the p-values, which are as follows: ns (not significant), * (p < 0.05), ** (p < 0.01), *** (p < 0.001), and **** (p < 0.0001).

## 3 Results and discussion

### 3.1 The combination of the *eyeless* promoter and *u* ^*S*^ *-Cas9* is suitable for gene disruption in adult eyes

**FIGURE 1:**
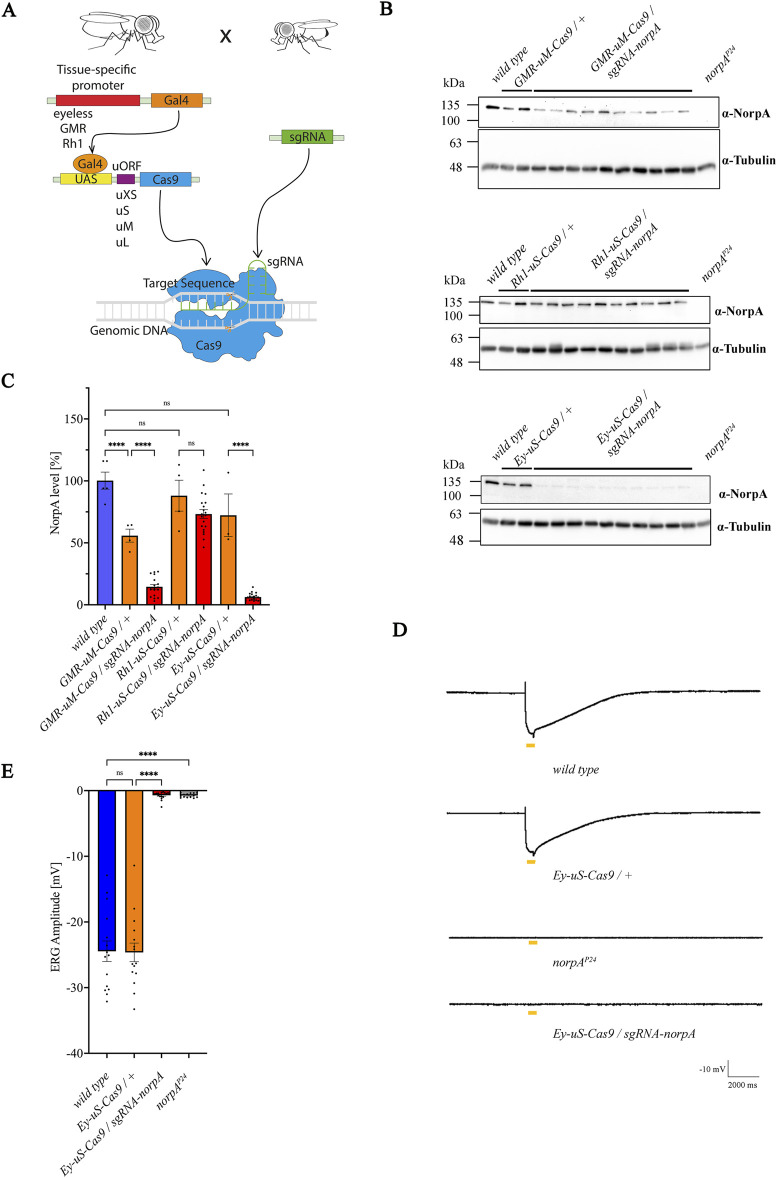
Combination of the *eyeless* promoter and *u* ^*S*^ *-Cas9* leads to the most efficient knock-out of *norpA*. **(A)** Scheme for the establishment of the tsCRISPR system in *Drosophila* eyes. **(B)** Immunoblot analysis assessing NorpA levels in various tissue specific *norpA* knock-outs. Genotypes of the driver constructs containing *GMR*, *Ey* or *Rh1* promoters recombined with different *Cas9* upstream variants are indicated above the panels. Single head extracts of 1–2 days old flies were analyzed and NorpA was detected by an α-NorpA antibody. α-Tubulin antibodies served as loading control. All lines were heterozygous for the eye specific promoter and *Cas9*. Molecular weight markers (kDa) are indicated on the left of each blot. **(C)** Quantification of the immunoblots shown in **(B)**. Statistically significant differences in panel C (n = 3-5 for controls; n = 20 for knock-out flies) were analyzed by a one-way ANOVA test with Bonferroni correction (**** p < 0.0001, ns not significant). Error bars: SEM. NorpA signals were normalized to the tubulin signal and the NorpA level of the wild type was set to 100%. **(D)** Electroretinogram recordings to assess the knock-out efficiency of NorpA in the indicated genotype of 1–2 days old dark-adapted flies illuminated with one orange light pulse of 500 msec. **(E)** Quantification of the electroretinogram amplitudes shown in **(D)**. Statistically significant differences in panel D (n = 15–21) were analyzed by a one-way ANOVA test with Bonferroni correction (**** p < 0.0001, ns not significant). Error bars: SEM.

Recent studies have highlighted the potential of tsCRISPR in *Drosophila*, using fly lines with sgRNAs and driver lines expressing the UAS activator Gal4, along with a Cas9 coding sequence regulated by an UAS promoter (Meltzer et al., 2019; Port et al., 2020; Meltzer et al., 2022). One of the strategies is that Cas9 expression levels are controlled by an uORF (upstream open reading frame) derived from the *GFP* sequence, whose length inversely modulates Cas9 protein levels. Various Cas9 constructs (*u* ^*XS*^-, *u* ^*S*^-, *u* ^*M*^-, *u* ^*L*^-Cas9 and *u* ^*XL*^-Cas9) exist, each with a sequence of disparate uORF length at the 5′region (*u* ^*XS*^ the shortest, *u* ^*XL*^ the longest), to yield distinct Cas9 protein levels. It has been demonstrated, that a shorter uORF corelates to a higher Cas9 expression, while a longer uORF leads to lower Cas9 levels, suggesting that a precise modulation of Cas9 activity in *Drosophila* tissue is possible (Port et al., 2020). CRISPR-mediated mutagenesis of target genes requires sufficient Cas9 activity, however a constitutive vector based expression of sgRNA-Cas9 complexes can cause non-specific mutagenesis and potentially cell toxicity, observable in various systems (Dow et al., 2015; Port and Bullock, 2016; Wu et al., 2014; Yang et al., 2018). In *Drosophila*, strong drivers may also induce high levels of Cas9 expression which may induce apoptosis even in the absence of gRNAs (Huynh et al., 2018; Poe et al., 2019; Port et al., 2014; Xue et al., 2014). Since Cas9 expression is the lowest in the *u* ^*XL*^-construct and highest in the *u* ^*XS*^-construct, it is crucial to attain a fine balance of sufficient Cas9 expression to mediate a successful on-target gene editing while avoiding cytotoxicity. To accomplish this, we have recombined various eye-specific promoter-Gal4 lines *glass multimer reporter (GMR)*, the *eyeless (Ey)* or the *rhodopsin* (*Rh1*) (Freeman, 1996; Quiring et al., 1994; Song et al., 2000) with *UAS-Cas9* variants to induce site directed mutagenesis in target genes, aiming to create null mutants of desired proteins in photoreceptors (Figure 1A). We focused particularly on constructs with *u* ^*S*^- and *u* ^*M*^-sized uORFs as these were shown to be most efficient with high on-target activity and low toxicity. We tested six eye-specific promoter *Gal4-UAS-Cas9* lines for gene editing efficiency and photoreceptor degeneration: *GMR > Gal4-UAS-u* ^*M*^ *-Cas9 (GMR-uM-Cas9)*, *Ey > Gal4-UAS-u* ^*M*^ *-Cas9 (Ey-uM-Cas9)*, *Ey > Gal4-UAS-u* ^*S*^ *-Cas9 (Ey-uS-Cas9)*, *Ey > Gal4-UAS-u* ^*XS*^ *-Cas9 (Ey-uXS-Cas9)*, *Rh1 > Gal4-UAS-u* ^*M*^ *-Cas9 (Rh1-uM-Cas9)* and *Rh1 > Gal4-UAS-u* ^*S*^ *-Cas9 (Rh1-uS-Cas9)*. The newly generated driver constructs were crossed to a *norpA-sgRNA* line to induce the knock-out of the photoreceptor specific *norpA* (no receptor potential A) gene*,* an essential component of the visual signal transduction cascade, devoid of an electroretinogram (ERG) response (Bloomquist et al., 1988; Ostroy and Pak, 1974). Knock-out efficiency of *norpA* was tested by immunoblot analysis of proteins extracted from single fly heads using α-NorpA antibodies (Figures 1B, C; Supplementary Figure S1A). As expected, we observed variations in NorpA levels among the different driver constructs. The combination of *GMR > Gal4* with *u* ^*M*^ *-Cas9* resulted in an incomplete NorpA knock-out while the *Rh1 > Gal4* driver line used here (in combination with *u* ^*S*^ *-Cas9 *or *u* ^*M*^-Cas9) showed no knock-out at all (Figures 1B, C; Supplementary Figures S1A, B). It would be interesting to see if other, perhaps non-commercially available *Rh1* drivers would perform at a higher efficiency. In case of the *GMR-uM-Cas9* construct, the incomplete knock-out may suggest a decreased efficiency of the driver construct. Previous research with two different *GMR > Gal4* insertion lines driving the expression of an *UAS-GFP* transgene not only showed variance in the robustness of expression but also tissue specificity, suggesting that the location of the *GMR > Gal4* transgene may differentially influence the phenotype of the same gene (Ray and Lakhotia, 2015). Eyeless is expressed very early in the eye development prior to photoreceptor determination in the third instar larva (Halder et al., 1995) and is also expressed in neurons (Callaerts et al., 2001). Surprisingly, a *norpA* knock-out was not observed with the *Ey-uM-Cas9* driver (Supplementary Figures S1A, B), therefore, we recombined *Ey > Gal4* with *uS-Cas9*. This time we observed a relatively high driver efficiency with a far more significant (average of 93%, relative to the no-sgRNA control) albeit not complete knock-out of *norpA* in all of the *Ey-uS-Cas9* and *Ey-uXS-Cas9* lines we tested (Figures 1B, C; Supplementary Figures S1A, B). To exclude the possibility of photoreceptor degeneration observed with some drivers (Bollepalli et al., 2017; Kramer and Staveley, 2003), we assessed photoreceptor functionality by ERG measurements (Figures 1D, E). *Ey-uS-Cas9/+* did not have a detrimental impact on photoreceptor function, while flies transheterozygous for the *Ey-uS-Cas9*/*sgRNA-norpA* construct demonstrated an average amplitude of only −0.7 mV, thus confirming the efficiency of the driver construct and corroborating the findings of the immunoblot analysis. Our results indicate that *Ey-uS-Cas9* is most suitable for inducing an efficient tsCRISPR mediated gene disruption in *Drosophila* eyes without leading to a degeneration of the photoreceptor cells and therefore, it was used for subsequent experiments. A drawback of driver lines using the *eyeless* promoter as compared, for example, with the *Rh1* promoter is its early and relative broad expression during development. The *eyeless* driver expresses Gal4 during eye development (Halder et al., 1995) and in neurons (Callaerts et al., 2001). *Eyeless* transcripts are detected in the eye disc primordia during embryogenesis and later in the developing eye imaginal discs during all early larval stages (Quiring et al., 1994). The expression of *eyeless* affects not only *Drosophila* eye development, but also developing neurons. This may result in lethality when essential genes are knocked out by this approach. Indeed, we obtained no offspring when the *Ey-uS-*Cas9 driver line was crossed with certain *Rab sgRNA* lines (see next chapter). Yet, an *eyeless* driver line has previously been used successfully to achieve a CRISPR-based tissue specific knock-out of target genes expressed in the eye and has proven to be more efficient than the RNAi-mediated knock-out of the same genes (Trivedi et al., 2020).

### 3.2 Several Rab proteins are involved in TRPL recycling

**FIGURE 2:**
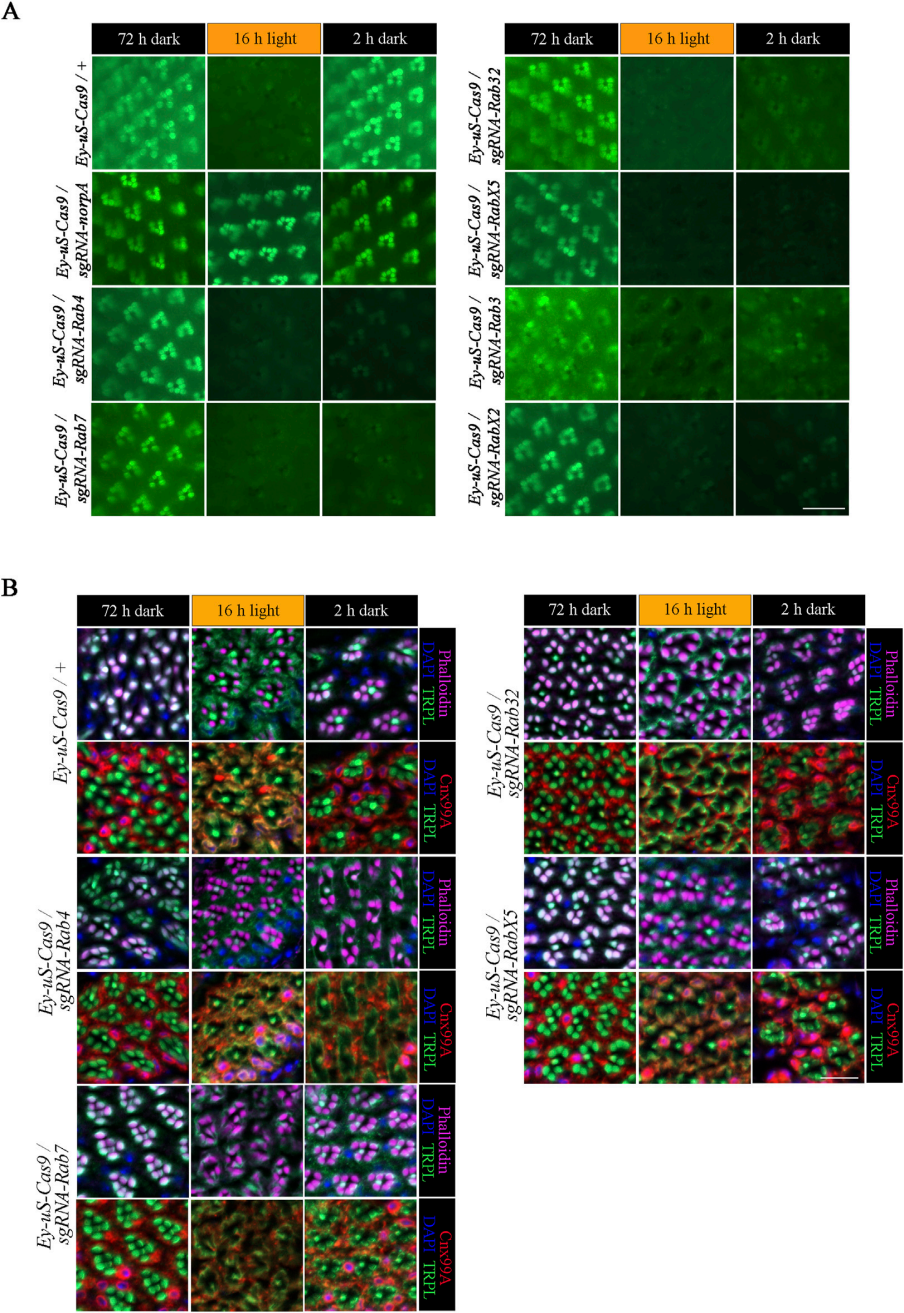
tsCRISPR mediated knock-out of specific Rab proteins results in a TRPL recycling defect. Of the 22 Rab proteins screened, 6 showed strong defects in TRPL recycling. **(A)** Water immersion micrographs showing TRPL::eGFP localization in *Drosophila* eyes of *Ey-uS-Cas9-TRPL::eGFP/+* and of *Ey-uS-Cas9-TRPL::eGFP/sgRNA-norpA* control flies, as well as the indicated *Rab* CRISPR mutants. Flies were kept in the dark for 72 h after eclosure, were exposed to orange light for 16 h and were subsequently returned to darkness for another 24 h. Scale bar: 20 µm. **(B)** Localization of TRPL in *Ey*-*uS-Cas9/+* control flies and *Rab4, Rab7, Rab32* and *RabX5* CRISPR mutants in d, d-l, and d-l-d conditions. Flies were dark-adapted for 72 h (d), subsequently exposed to orange light for 16 h (d-l), and were subsequently returned to darkness for another 2 h (d-l-d). Cross sections through ommatidia were probed with α-TRPL antibodies and α-Cnx99A antibodies, as indicated. Rhabdomeres were visualized using phalloidin and nuclei were stained with DAPI. Scale bar: 10 μm.

Rab proteins are studied for their role in recycling of diverse proteins in multiple systems (Zhang et al., 2022), however, their role in *Drosophila* TRPL recycling still needs to be elucidated. To identify Rab proteins involved in TRPL recycling, we employed tsCRISPR/Cas9 mutagenesis to induce disruption of *Rab* genes. Of the 26 commercially available *Rab-sgRNA* fly lines from Bloomington and Vienna *Drosophila* stock centers, 22 were tested with respect to TRPL recycling defects. Flies carrying sgRNAs directed against *Rab1, Rab5, Rab6* and *Rab11* produced no viable offspring when crossed with the *Ey-uS-Cas9* driver construct. Since the *eyeless* driver expresses Gal4 during eye development and in neurons these findings may indicate, that these Rabs are essential during neuronal development. We assayed the expression of TRPL::eGFP signal in flies transheterozygous for *Ey-uS-Cas9* and the specific *Rab-sgRNA* using WI-mediated fluorescent microscopy, an efficient technique to obtain quick initial results (Wagner et al., 2022b). Rab gene disruptions analyzed by WI microscopy showed no obvious signs of disturbed rhabdomere formation or rhabdomere degeneration except for *RabX1*, which revealed diffused TRPL::eGFP labeling of rhabdomeres and the presence of many TRPL::eGFP positive vesicles in dark-adapted flies (Supplementary Figure S2). Disruption of *Rab10* resulted in an internalization defect (Supplementary Figure S2). Since the detection of eGFP signal by WI-microscopy in red-eyed flies is compromised due to pigmental interference, we attempted to reduce the amount of pigmentation by RNAi knock-down targeting the mini *white* gene present in each of the transgenes. This approach resulted in flies with white to light orange eye pigmentation, which ameliorated imaging by fluorescent microscopy. It has to be noted, that in the WI microscopy assay, even slight levels of orange pigmentation may still partially quench TRPL::eGFP fluorescence in the eye, which could interfere with proper quantification. Therefore, we refrained from quantifying the fluorescence signals and resorted instead to qualitative evaluation by visual assessment. To analyze TRPL translocation in *Rab* mutants, test flies were subjected to different illumination conditions. Prior to the analysis, flies were kept in darkness for 72 h (d condition), were subsequently exposed to orange light for 16 h (d-l condition) and were dark-adapted once again for 24 h (d-l-d condition). *Ey-uS-Cas9-TRPL::eGFP/+* was used as a suitable wild type control, while knock-outs of *norpA* and *vps35* were utilized as examples for a TRPL internalization defect and for a recycling defect, respectively. The complete failure of TRPL::eGFP internalization in *Ey-uS-Cas9-TRPL::eGFP/sgRNA-norpA* confirmed the efficiency of this driver construct once more (Figure 2A). As a positive control for a TRPL recycling defect, we employed a tsCRISPR/Cas9-mediated knock-out of *vps35* (Vacuolar protein sorting 35), a component of the retromer complex and needed for TRPL recycling (Wagner et al., 2022a). Disruption of *vps35* resulted in reduced rhabdomeral TRPL::eGFP compared to wild type in the d-l-d condition, indicating a TRPL recycling defect (Supplementary Figure S2).

We had previously studied TRPL internalization defects using dominant negative Rab (RabDN) proteins and found that Rab1DN, Rab4DN, Rab5DN, Rab10DN, Rab19DN, and RabX4DN resulted in failures of TRPL internalization in the d-l condition (Oberegelsbacher et al., 2011). *Rab1* and *Rab5* could not be studied here, as tsCRISPR knock-out produced no viable offspring. tsCRISPR knock-out of *Rab10* resulted in a strong internalization defect in accordance with previous data. Knock-out of *Rab4*, *Rab19*, and *RabX4* showed partial, if any, internalization defects that were not as prominent as observed with RabDN mutants. This discrepancy may result from shorter illumination times used by Oberegelsbacher et al. (6 h orange light illumination). In addition, off-target effects of RabDN constructs or a failure of knock-out with some sgRNA can not be excluded. In the present study we focus on TRPL recycling defects after TRPL was fully internalized by 16 h of orange light illumination.

**FIGURE 3:**
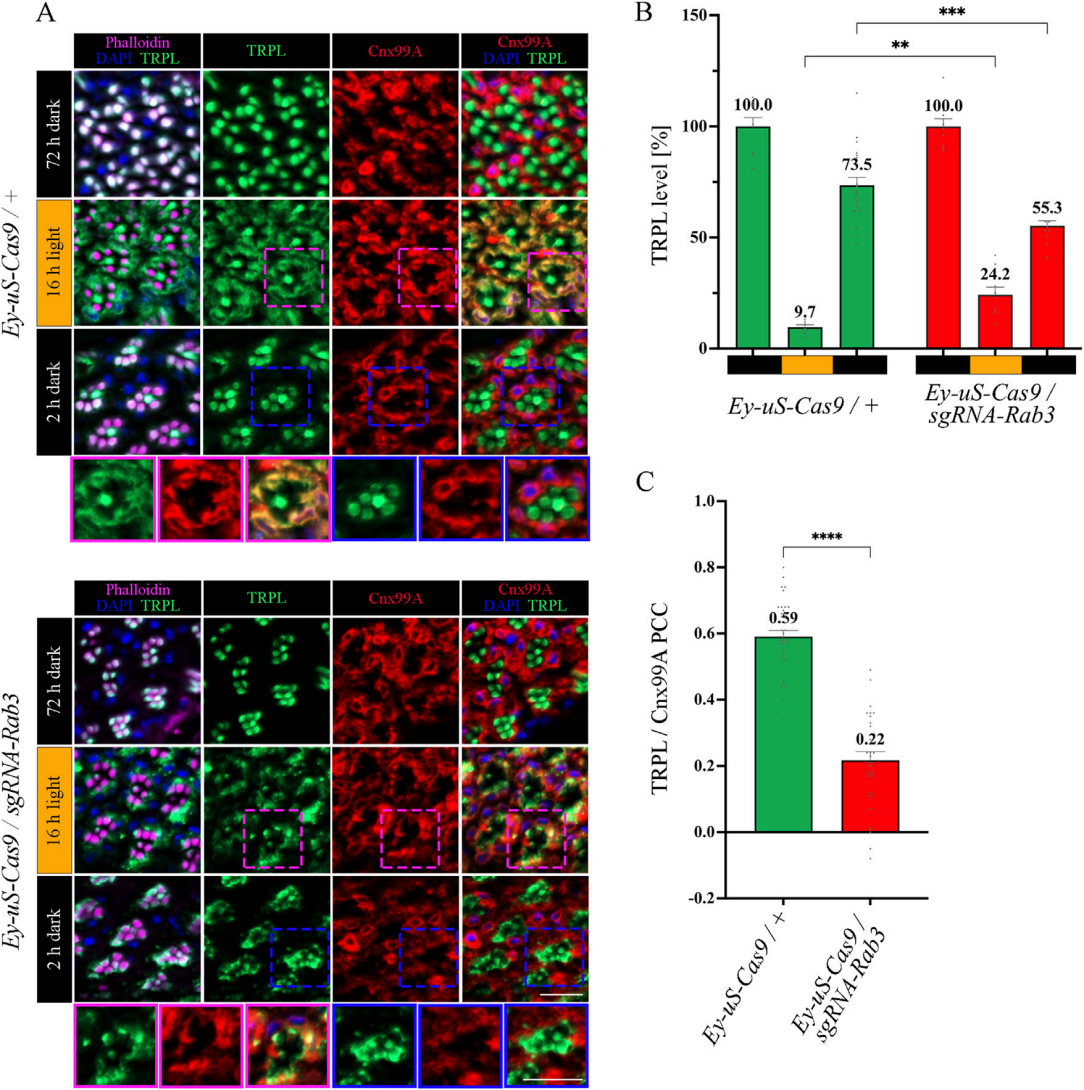
*Rab3* gene disruption hinders TRPL transport to the ER. **(A)** Localization of TRPL in *Ey-uS-Cas9/+* control flies and *Rab3* CRISPR mutants in d, d-l, and d-l-d conditions. Flies were dark-adapted for 72 h (d), then exposed to orange light for 16 h (d-l), and were subsequently returned to darkness for another 2 h (d-l-d). Cross sections through ommatidia were probed with α-TRPL antibodies and α-Cnx99A antibodies, as indicated. Rhabdomeres were visualized using phalloidin and nuclei were stained with DAPI. Images at the bottom of each panel show a magnification of the indicated areas above. Scale bar: 10 μm. **(B)** Quantification of fluorescence signals of TRPL in the rhabdomeres. Signals in R1-R6 rhabdomeres were normalized to the signal of R7 rhabdomeres and the mean of values obtained after the initial dark adaptation was set to 100%. Statistically significant differences analyzed by a two-way ANOVA calculation with Bonferroni correction are indicated (** p < 0.01, *** p < 0.001). Error bars: SEM (n = 3–7). **(C)** Colocalization of TRPL with the ER marker Cnx99A after 16 h of orange light illumination. Colocalization was assessed using Pearson correlation. The TRPL channel was chosen for the selection of the areas to be quantified. Statistically significant differences analyzed by an unpaired t-test (**** p < 0.0001) Error bars: SEM (n = 5–6).

Of all the *Rab* gene disruptions analyzed by WI microscopy *Rab3*, *Rab4*, *Rab7*, *Rab32*, *RabX2* and *RabX5* displayed the strongest TRPL recycling defects (Figure 2; Supplementary Figure S2). To investigate the defects observed in these mutants, we performed IHC with antibodies against TRPL and the ER marker calnexin (Cnx99A) to observe colocalization of TRPL at the ER and stained with phalloidin to visualize rhabdomeres (Figure 2B for Rab4, Rab7, Rab32, RabX5; Figure 3A for Rab3; Figure 6A for RabX2). TRPL was present in the rhabdomeres of *Rab4*, *Rab7*, *Rab32* and *RabX5* mutants after 72 h of initial dark adaptation. Little or no colocalization of TRPL with Cnx99A was observed in *Rab7* and *Rab32* mutants in the d-l condition, but a large amount of TRPL was recycled in the d-l-d condition in these mutants (Figure 2B). In the d-l-d condition TRPL was also detected outside the rhabdomere in *Rab7* knock-out flies. In contrast to *Rab32* and *Rab7* mutants, TRPL and Cnx99A were colocalized in *Rab4* mutants, but only partial redistribution of TRPL to R1-6 rhabdomeres was observed after the second dark adaptation, indicating a stronger recycling defect (Figure 2B). TRPL recycling in *RabX5* mutants was similar to the control and the TRPL recycling defect observed by WI microscopy was not detectable by IHC. We found the recycling defect phenotype of *Rab3* and *RabX2* the most interesting, therefore we focused our attention on the analysis of these mutants.

### 3.3 Rab3 is needed for TRPL recycling at the late endosome

The function of Rab3 was initially described in the context of the regulation of synaptic vesicles fusion (Takai et al., 1996; Encarnação et al., 2016; Lledo et al., 1994; Marsh et al., 2007; Oishi et al., 1998; Schlüter et al., 2004; Südhof, 2004). However, a recent study in human embryonic kidney (HEK) cells showed that in the absence of Rab3, lipid raft proteins accumulate in late endosomes, leading to recycling defects in these cells (Diaz-Rohrer et al., 2023). We aimed to investigate the nature of the TRPL recycling defects, observed during WI microscopy in *Rab3* knock-out mutants, in more detail with IHC analysis. The flies were subjected to an illumination protocol analogous to the protocol used for the WI microscopy with the exception of the second dark period being shortened to 2 h, which is sufficient to achieve complete recycling of TRPL to the rhabdomere without a notable contribution of newly synthesized TRPL in wild type flies (Wagner et al., 2022a) (Supplementary Figure S3). Cross- and longitudinal cryosections of compound eyes of *Rab3* knock-out mutants and their corresponding control flies were treated with α-TRPL and α-Cnx99A antibodies. Quantification of TRPL levels in rhabdomeres was analyzed based on TRPL immunofluorescence intensity in rhabdomeres R1-6 and was normalized to the signal intensity from rhabdomere R7. The R7 rhabdomere contains a UV-sensitive rhodopsin and thus the R7 cell does not show TRPL translocation upon orange light stimulation. In a previous study, Wagner et al. demonstrated that in wild type flies TRPL colocalized with the ER marker Cnx99A after orange light illumination, as revealed by a high Pearson correlation coefficient (PCC) of 0.7 for TRPL and Cnx99A (Wagner et al., 2022a). A similar PCC of 0.59 for TRPL and Cnx99A was observed here for the control fly in the d-l condition.

In *Rab3* knock-out mutants, TRPL recycling in the d-l-d condition was affected. TRPL signals were detected partly in the stalk membrane, predominantly at the rhabdomere base and in the cell body, rather than in the rhabdomere (Figure 3A). Quantification of TRPL signals in the rhabdomere confirmed a significant reduction of TRPL level (55.3%) in the rhabdomeres of these mutants, compared to control flies (73.5%) (Figure 3B). More prominently, the *Rab3* mutation affected transport of TRPL to the ER in the d-l condition. As indicated by a PCC of 0.22 (Figure 3C), the *Rab3* mutation resulted in a disruption of the colocalization of TRPL with Cnx99A, similar to that observed previously in the *vps35* ^*MH20*^ mutant (Wagner et al., 2022a). To enrich experimental evidence for the phenotype resulting from the tsCRISPR-mediated disruption of *Rab3*, the same IHC experiment was repeated under the same illumination conditions with a *Rab3 null* mutant (*Rab* ^*Rup*^) (Graf et al., 2009). The results were found to be similar (Supplementary Figure S4). In both *Rab3* mutants (*Rab3Rup* and *Rab3 CRISPR*), TRPL did not translocate to the ER and only partially returned to the rhabdomere (Figures 3A–C; Supplementary Figure S4). This suggests that Rab3 is required for the light-dependent transport of TRPL to the ER.

**FIGURE 4:**
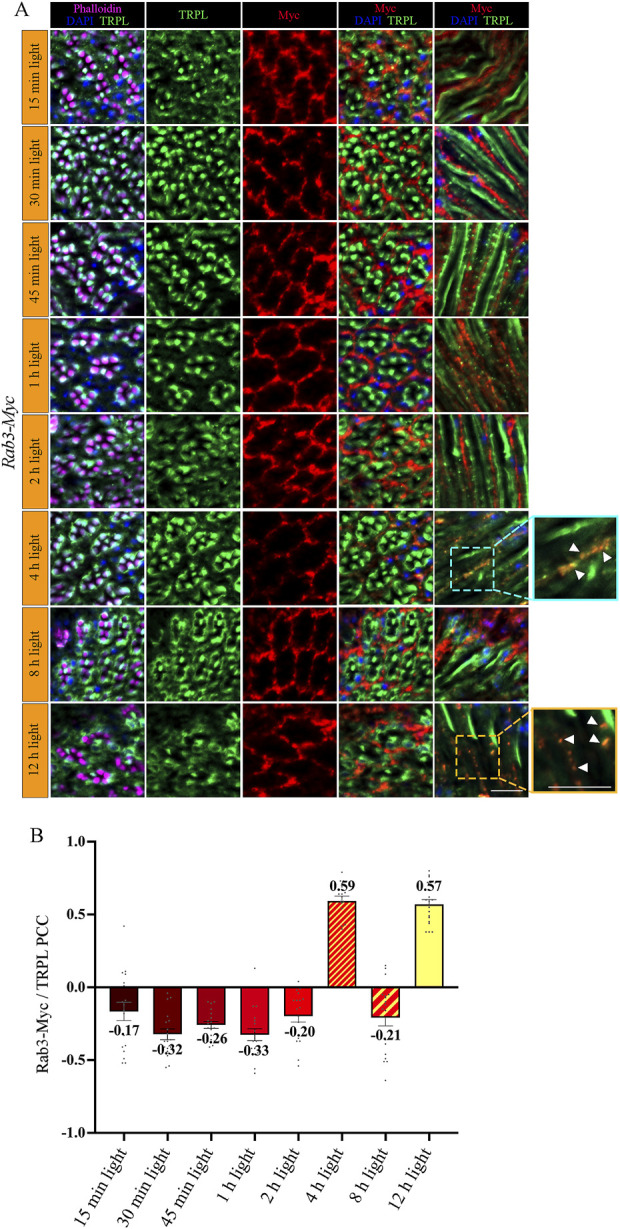
Rab3-Myc is colocalized with TRPL after 4 h and 12 h of light adaption. **(A)** Analysis of the colocalization of TRPL and Rab3-Myc by immunohistochemistry in cross- and longitudinal-sections through ommatidia at different time points of orange light illumination. Flies were initially kept in the dark for 1 day and were then exposed to orange light for 15 min, 30 min, 45 min, 1 h, 2 h, 4 h, 8 h, and 12 h. TRPL was detected with an α-TRPL antibody and Rab3-Myc with an α-Myc antibody. Rhabdomeres were visualized with phalloidin and nuclei with DAPI. The images on the right illustrate an enlargement of the marked areas. Arrowheads indicate colocalization of TRPL with Rab3-Myc at time points 4 h and 12 h. Scale bar: 10 μm. **(B)** Quantification for colocalization of Rab3-Myc with TRPL after 15 min, 30 min, 45 min, 1 h, 2 h, 4 h, 8 h and 12 h of orange light illumination. The TRPL channel was chosen for the selection of the areas to be quantified. Colocalization was assessed using Pearson correlation. Error bars: SEM (n = 3).

**FIGURE 5:**
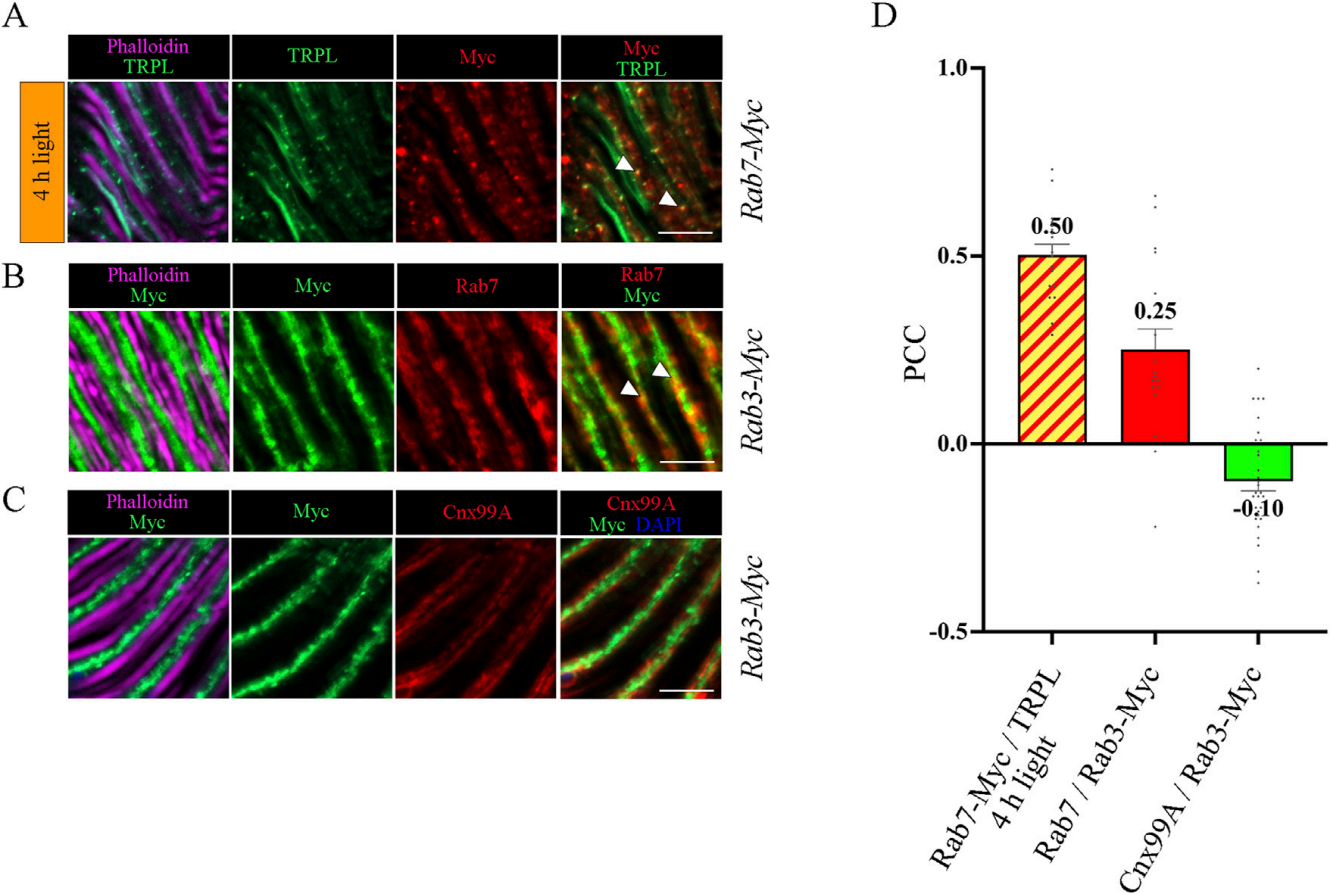
Colocalization analysis of TRPL, Rab3 and Rab7. **(A)** Colocalization of TRPL and Rab7-Myc positive vesicles in longitudinal sections of *Rab7-Myc* ommatidia. Flies were initially kept in the dark for 1 day and were then exposed to orange light for 4 h. TRPL was labeled with an α-TRPL antibody, Rab7-Myc was labeled with an α-Myc antibody. Rhabdomeres were visualized with phalloidin. Arrowheads indicate colocalization of TRPL with Rab7-Myc at time point 4 h of orange light illumination. Scale bar: 10 μm. **(B)** Colocalization of Rab7 and Rab3-Myc in longitudinal cryosections of *Rab3-Myc* ommatidia. Rab7 was labeled with an α-Rab7 antibody, Rab3-Myc was labeled with an α-Myc antibody. Rhabdomeres were visualized using phalloidin. Arrowheads indicate colocalization of Rab7 with Rab3-Myc. **(C)** Colocalization of Rab3-Myc and Calnexin in longitudinal cryosections of *Rab3-Myc* ommatidia. Calnexin was labeled with an α-Cnx99A antibody, Rab3-Myc was labeled with an α-Myc antibody. Rhabdomeres were visualized using phalloidin and nuclei using DAPI. Scale bar: 10 μm **(D)** Quantification of colocalization of TRPL with Rab7-Myc after 4 h of orange light illumination. The TRPL channel was chosen for the selection of the areas to be quantified. Quantification of colocalization of Rab7 with Rab3-Myc, and Cnx99A with Rab3-Myc. The Rab3-Myc channel was chosen for the selection of the areas to be quantified. Colocalization was assessed using Pearson correlation. Error bars: SEM (n = 3–5).

To investigate the temporal relationship between TRPL and Rab3 during TRPL transport to the ER, colocalization studies between Rab3-Myc and TRPL were conducted at various time points after orange light illumination (15 min, 30 min, 45 min, 1 h, 2 h, 4 h, 8 h, 12 h). Cross- and longitudinal cryosections of transgenic *Drosophila* eyes of flies expressing a Myc-tagged Rab3 were treated with α-Myc and α-TRPL antibodies. Colocalization of Rab3-Myc and TRPL was observed to be significant after 4 h (PCC of 0.59) and 12 h (PCC of 0.57) (Figures 4A, B). It is noteworthy that no colocalization was observed between Rab3-Myc and TRPL after 8 h of orange light illumination (PCC of −0.21). This indicates the existence of two distinct subtypes of Rab3 compartments containing TRPL. TRPL was previously shown to colocalize with the early endosome after 2 h of light adaption (Wagner et al., 2022a), and here we find that TRPL is present at the late endosome after 4 h of light adaptation by showing its colocalization with the late endosome marker Rab7-Myc (PCC of 0.5) (Figures 5A, D). We proceeded to examine the colocalization between the late endosomal marker Rab7 and Rab3-Myc. By immunohistochemistry, we demonstrated partial colocalization of Rab7 and Rab3-Myc (average PCC of 0.25) (Figures 5B, D). However, it should be noted that these PCC values showed a very high variance (from 0.66 to −0.22), which may indicate that parts of the Rab7 positive late endosome contain Rab3-Myc while other parts do not. Upon 12 h of illumination with orange light, TRPL is found to colocalize with Cnx99A. Given that Rab3-Myc is also colocalized with TRPL after 12 h (Figures 4A, B), we postulated that Rab3 may localize at the ER. Consequently, we investigated the colocalization between Rab3-Myc and Cnx99A. To our surprise, we did not observe any colocalization between Rab3-Myc and Cnx99A (PCC of −0.10) (Figures 5C, D). Therefore, it is possible that some of the recycled TRPL may colocalize with an unknown subtype of Rab3 vesicles instead of Cnx99A. These findings collectively indicate that Rab3 is essential for the transport of TRPL from the plasma membrane to the ER in late endosomes.

### 3.4 Disruption of *RabX2* leads to an accumulation of TRPL at the trans-Golgi

Information about the subcellular localization and function of *Drosophila* RabX2 is scarce. Studies on the unicellular parasite *Trypanosoma brucei* report that RabX2 is located at the Golgi (Field et al., 2000; Natesan et al., 2009). Field et al. (2000) showed RabX2 (TbRAB31) association with the Golgi by electron microscopy (EM) and using two established markers of the Golgi. A second study in the same organism by Natesan et al. (2009) showed subcellular localization of RabX2 at the Golgi complex, but could not discern a major role of RabX2 in intracellular transport. For *Drosophila,* RabX2 has been predicted to be localized at the Golgi (Gaudet et al., 2011) but this this has not yet been experimentally verified.

**FIGURE 6:**
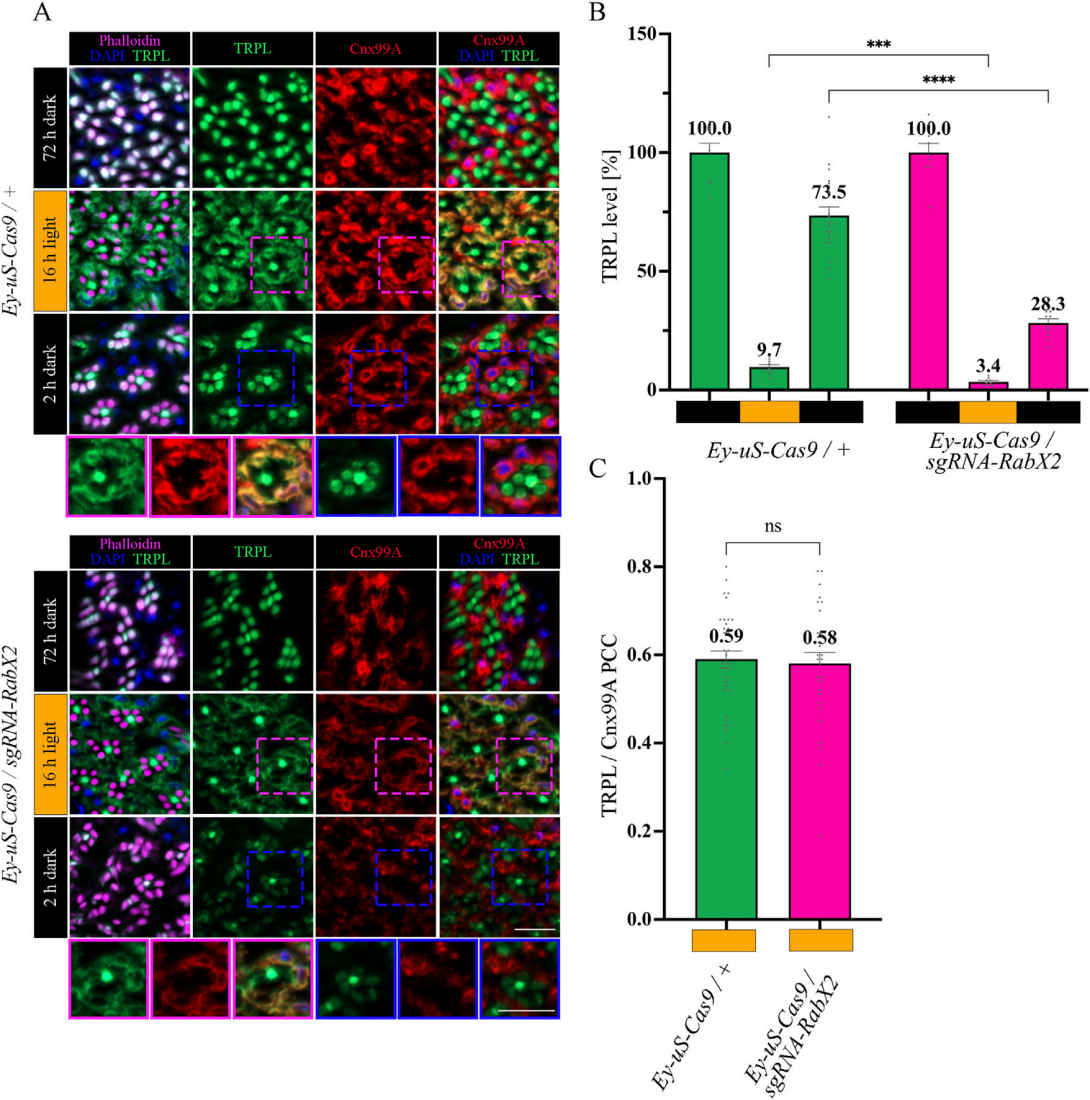
Recycling of TRPL to the rhabdomere depends on RabX2. **(A)** Localization of TRPL in eye cross-sections of *Ey*-*uS-Cas9/+* control flies and *RabX2* CRISPR mutants in d, d-l, and d-l-d light conditions. Flies were dark-adapted for 72 h after eclosure, then exposed to orange light for 16 h and were subsequently returned to darkness for another 2 h. Sections were probed with α-Cnx99A and α-TRPL antibodies. Rhabdomeres were stained with phalloidin and nuclei with DAPI. Images at the bottom show magnifications of the indicated areas above. Scale bar: 10 μm. **(B)** Quantification of TRPL content in rhabdomeres. Signals in R1-R6 rhabdomeres were normalized to R7 signals and values obtained after the initial dark adaptation were set to 100%. Statistically significant differences analyzed by a two-way ANOVA calculation with Bonferroni correction are indicated (**** p < 0.0001, *** p < 0.001) Error bars: SEM (n = 3–7). **(C)** Colocalization of TRPL with the ER marker Cnx99A after 16 h of orange light illumination. Colocalization was assessed using Pearson correlation. The TRPL channel was chosen for the selection of the areas to be quantified. Statistically significant differences as analyzed by an unpaired t-test (ns not significant) Error bars: SEM (n = 5–6).

**FIGURE 7:**
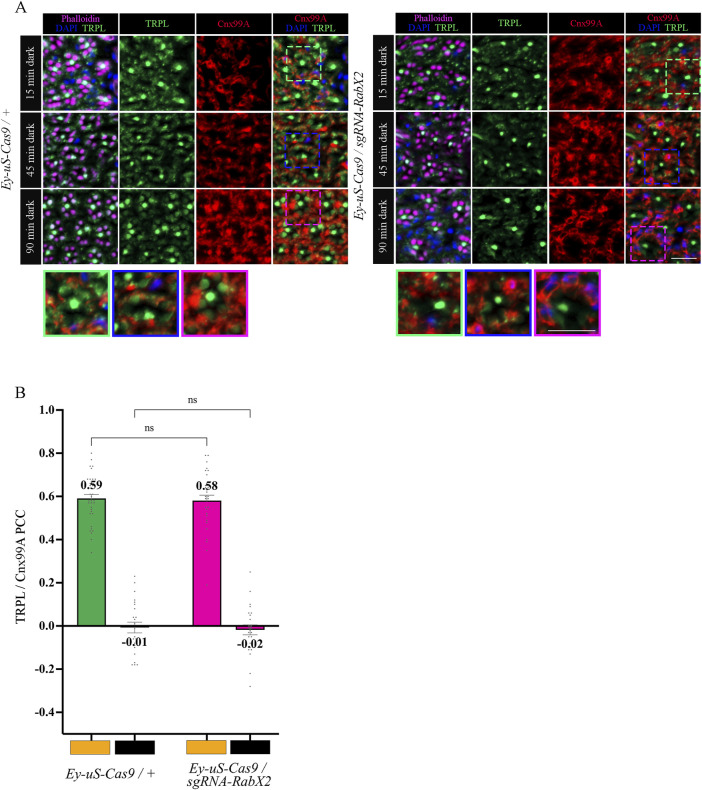
In *RabX2* mutants TRPL is released from the ER after 15 min dark adaptation but does not reach the rhabdomere after 90 min. **(A)** Localization of TRPL in cross-sections of *Ey-uS-Cas9/+* control and *RabX2* CRISPR mutant ommatidia. After 16 h of orange light exposure, flies were dark-adapted for 15 min, 45 min, and 90 min. Sections were probed with α-Cnx99A and with α-TRPL antibodies. Rhabdomeres were stained with phalloidin and nuclei with DAPI. Images at the bottom show magnifications of the indicated areas above. Scale bar: 10 μm. **(B)** Colocalization of TRPL with the ER marker Cnx99A after 16 h of orange light illumination and 15 min of dark adaptation. Colocalization was assessed using Pearson correlation. The TRPL channel was chosen for the selection of the areas to be quantified. Statistically significant differences as analyzed by a two-way ANOVA calculation with Bonferroni correction are indicated (ns not significant) Error bars: SEM (n = 4–5).

The trans-Golgi network was shown to be important for protein recycling (Appenzeller-Herzog and Hauri, 2006; Capitani and Sallese, 2009; Cottam and Ungar, 2012). Previous research indicated the ER rather than the trans-Golgi as a reservoir for TRPL proteins deemed for recycling, however the recycling route from the ER to the rhabdomere remains unknown (Wagner et al., 2022a). To verify the TRPL recycling defect observed in the WI analysis of the *RabX2* knock-out mutants (Figure 2A), we performed IHC analysis with α-TRPL and α-Cnx99A antibodies on cross- and longitudinal cryosections of 3-day-old flies and their corresponding controls. After 16 h of orange light exposure, TRPL levels in rhabdomeres decreased to approximately 9.7% in control flies and 3.4% in the mutants (Figures 6A, B). However, in contrast to *Rab3* knock-out mutants, where TRPL localization at the ER was strongly reduced under the same illumination conditions, *RabX2* knock-out mutants displayed a wild type TRPL localization at the ER (PCC of 0.58) (Figures 6A, C), indicating that RabX2 has no influence on TRPL transport to the ER. A subsequent dark adaptation for 2 h revealed that the TRPL amount in the rhabdomere was not completely replenished in *RabX2* knock-out flies (28.3%), when compared to control flies (73.5%) (Figures 6A, B). In wild type flies, TRPL is present in the rhabdomeres after d and d-1-d conditions and at the ER in d-l condition, and TRPL recycling from the ER to the rhabdomere is completed within 90 min (Wagner et al., 2022a). Although we detected nearly wild type levels of TRPL in rhabdomeres in the d conditon in *RabX2* knock-out mutants, reduced levels of TRPL in d-1-d condition, in combination with colocalization between TRPL and Cnx99A suggest that TRPL transport from the ER to the rhabdomere is compromised. To investigate whether RabX2 is required for the release of TRPL from the ER, we investigated TRPL localization in the *RabX2* knock-out mutants at different d-l-d time points (15, 45 and 90 min) during TRPL transportation from the ER. Cryosections of *RabX2* knock-out mutant eyes and their respective controls were treated with α-Cnx99A and α-TRPL antibodies and a TRPL-Cnx99A colocalization was quantified by Pearson correlation (Figures 7A, B). When compared to d-l condition, the 15 min d-1-d condition revealed a significant drop in the TRPL-Cnx99A colocalization in both, control (PCC 0.59 to −0.01) and *RabX2* knock-out mutants (PCC 0.58 to −0.02). These results show that the TRPL recycling defect in *RabX2* knock-out mutants is not a result of a failure to release TRPL from the ER, but rather its inability to return to the rhabdomeres, suggesting that TRPL is retained at other intracellular compartments.

**FIGURE 8:**
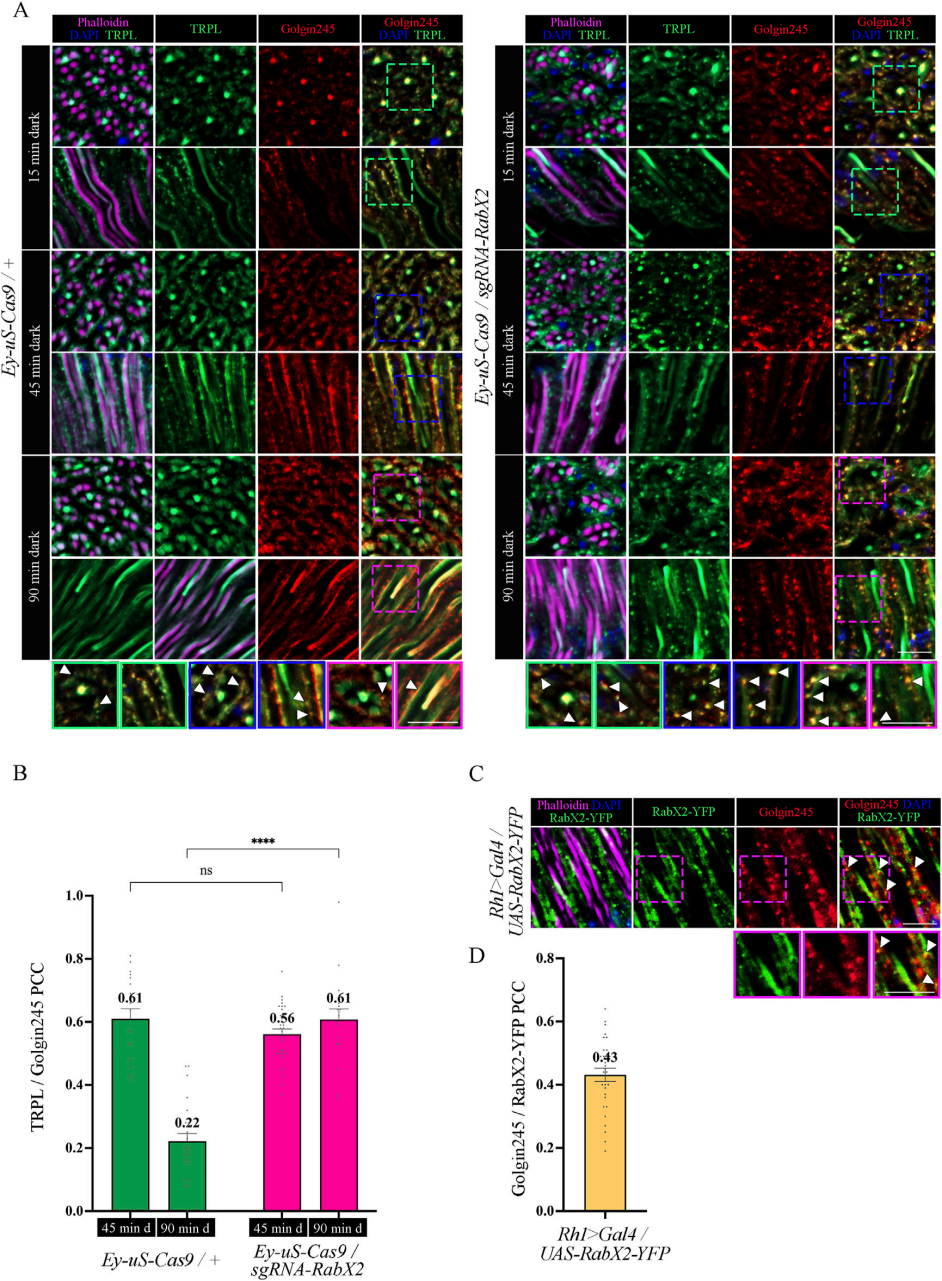
Knock-out of *RabX2* leads to TRPL accumulation at the trans-Golgi. **(A)** Localization of TRPL in eye cross and longitudinal -sections of *Ey-uS-Cas9/+* control flies and *RabX2* knock-out flies. After 3 days of dark adaption and 16 h of orange light exposure, flies were dark-adapted for 15 min, 45 min, and 90 min. Sections were probed with α-Golgin245 (labelling trans-Golgi) and α-TRPL antibodies. Rhabdomeres were stained with phalloidin and nuclei with DAPI. Images at the bottom show magnifications of the indicated areas above. Scale bar: 10 μm. **(B)** Colocalization of TRPL with Golgin245 after 45 min or 90 min of dark adaptation. Colocalization was assessed using Pearson correlation. The TRPL channel was chosen for the selection of the areas to be quantified. Statistically significant differences analyzed by a two-way ANOVA calculation with Bonferroni correction are indicated (**** p < 0.0001, ns not significant) Error bars: SEM (n = 3–5) **(C)** Localization of RabX2-YFP in eye longitudinal sections of *Rh1 > Gal4/UAS-RabX2-YFP* flies. Sections were probed with α-Golgin245 (labelling trans-Golgi) antibody. Rhabdomeres were stained with phalloidin and nuclei with DAPI. Images at the bottom show magnifications of the indicated areas above. Scale bar: 10 μm. **(D)** Quantification for colocalization of RabX2-YFP with Golgin245. The Golgin245 channel was chosen for the selection of the areas to be quantified. Colocalization was assessed using Pearson correlation. Error bars: SEM (n = 5).

Next, we analyzed the colocalization between TRPL and the trans-Golgi under different d-l-d conditions (15 min, 45 min, 90 min) by IHC. Therefore, we treated cross- and longitudinal cryosections of *RabX2* knock-out mutant eyes and their respective controls with antibodies against the trans-Golgi marker α-Golgin245 and α-TRPL. In the control, we observed that TRPL and Golgin245 colocalized after 45 min of d-l-d condition (PCC of 0.61), but after 90 min of d-l-d a very significantly reduced colocalization was observed (PCC of 0.22) (Figures 8A, B). Interestingly, strong colocalization was observed in *RabX2* knock-out mutants both after 45 min of d-l-d (PCC of 0.56) and after 90 min of d-l-d (PCC of 0.61) (Figures 8A, B). This suggests that RabX2 is required for the recycling of TRPL to the plasma membrane via the trans-Golgi network. In order to confirm the prediction that RabX2 is localized at the trans-Golgi, RabX2-YFP was expressed under the *Rh1* promoter. Subsequently, we investigated the colocalization between the trans-Golgi marker Golgin245 and RabX2-YFP. Partial colocalization of RabX2-YFP and Golgin245 was observed (PCC of 0.43) (Figures 8C, D). It is important to note, however, that the PCC values exhibit a considerable degree of variability (ranging from 0.64 to 0.19), which could suggest the presence of RabX2-YFP in some regions of the trans-Golgi apparatus, while other sections may lack this protein. In addition, we observed that the TRPL signals in the rhabdomeres of the *RabX2* mutant were significantly reduced when compared to control flies after two hours dark adaption (d-l-d condition; Figure 6B) and virtually no signal was detected in the cell body. This finding may suggest that a significant amount of TRPL is degraded rather than being recycled to the rhabdomere. In summary, our findings indicate that at least a portion of TRPL is recycled via the trans-Golgi where it accumulates in *RabX2* gene disruption flies and then becomes degraded. This presents a novel pathway for TRPL recycling with the trans-Golgi as a paramount compartment for retrograde TRPL transport.

## 4 Conclusion

**FIGURE 9:**
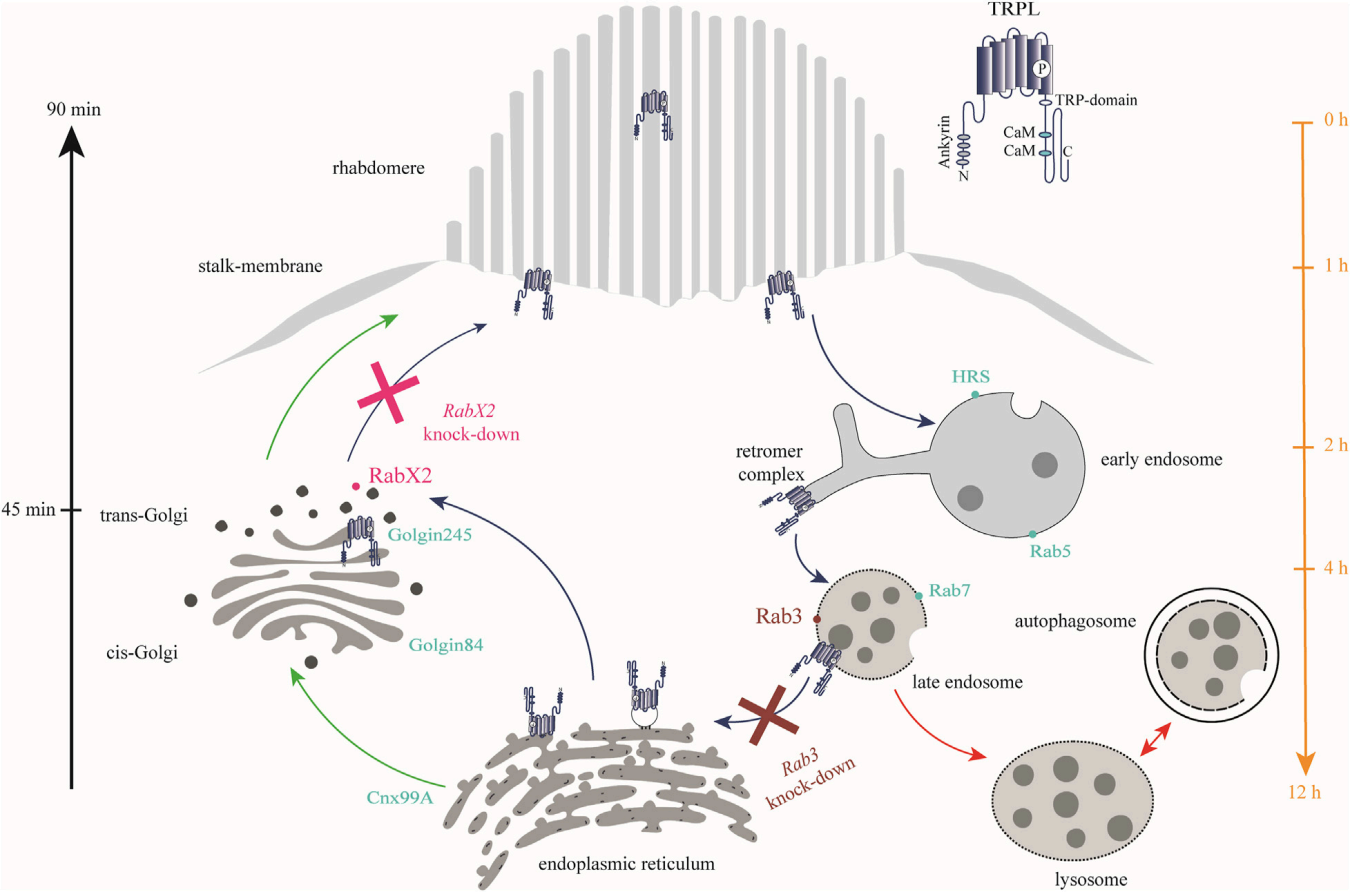
Rab3 and RabX2 are important components of TRPL recycling. In the dark, TRPL is localized to the rhabdomeric membrane and translocates to the endosomal network within 2 h after light adaptation. From there, it is transported to the ER within 12 h, where it is temporarily stored. Upon renewed dark adaptation, TRPL is transported from the ER via the trans-Golgi network back to the rhabdomeric membrane within 90 min. Anterograde transport due to *de novo* synthesis of TRPL occurs via the cis- and trans-Golgi networks (green arrows). A small fraction of internalized TRPL is degraded via the lysosomal degradation pathway (red arrows). Rab3 was identified at the late endosome and is essential for TRPL transport to the ER as well as for proper recycling (burgundy). In contrast, knock-out of *RabX2* resulted in accumulation of TRPL at the trans-Golgi (magenta). Marker proteins for the different membrane enclosed compartments are indicated by dots (turquoise).

In conclusion, Rab3 and RabX2 were shown to be important regulators of the TRPL recycling pathway. Upon knock-out of *Rab3*, a reduction in ER colocalization and TRPL recycling was observed. This was characterized by an accumulation of TRPL vesicles at the rhabdomeric base, stalk membrane, and cell body (Figures 3A–C; Figure 9). Colocalization between Rab3-Myc and TRPL was observed after 4 and 12 h of light adaptation (Figures 4A, B). Previous research in HeLa cells showed colocalization of Rab3 with Rab7 and suggested a role of Rab3 in recycling of proteins in Rab7 positive endosomes (Diaz-Rohrer et al., 2023). Partial colocalization between the late endosomal marker Rab7 and Rab3-Myc was observed, leading to the conclusion that Rab3 is partially localized to the late endosome (Figures 5A, D; Figure 9). Our study substantiates a role of Rab3 in the late endosome for proper recycling in the photoreceptor cells of the compound eyes of *Drosophila melanogaster*. Rab3 is a highly conserved gene with homologs in humans and vertebrate model organisms. In humans, Rab3 plays an important role in regulating the exocytosis of hormones and neurotransmitters in response to Ca^2+^-mediated signaling. (Li and Chin, 2003; Südhof, 2004). Mutations in members of the *Rab3* family are linked to neurodevelopmental diseases (Aligianis et al., 2006; Picker-Minh et al., 2014; Udayar et al., 2013). Our research is the first to demonstrate in an *in vivo* model system, that Rab3 is not only required for the regulation of synaptic vesicle fusion, but also for protein recycling. A similar phenotype, that shows no colocalization of TRPL and the ER marker Cnx99A was observed after knock-out of *Rab7*. It should be noted that in both mutants, although recycling was not as complete as in the control, a significant amount of TRPL could still be detected in the rhabdomere after the second dark adaptation. Similarly, knock-out of *Rab32* resulted in a reduction in ER colocalization, but TRPL was detected in the rhabdomere after the second dark adaptation. These findings may argue for an additional TRPL recycling pathway that does not involve its transport and storage at the ER and has yet to be elucidated.

Following *RabX2* knock-out, TRPL accumulates at the trans-Golgi and its transport back to the rhabdomeric membrane is hindered (Figures 8A, B; Figure 9). However, the defect is not the result of a hindered TRPL release from the ER, but rather from TRPL´s inability to return to the rhabdomeres. Although not investigated in detail here, a similar phenotype was observed after light and subsequent dark adaptation for *Rab4* knock-out. Both mutants exhibited native ER colocalization and markedly reduced TRPL recycling. Rab4 was identified at the recycling endosome, as previously reported (West et al., 2015). Given that a fusion between trans-Golgi compartments and recycling endosomes in retrograde transport has already been described in *Drosophila*, it is conceivable that TRPL recycling from the ER to the rhabdomeric membrane not only traverses the trans-Golgi, but also the recycling endosome (Fujii et al., 2020). This presents a novel pathway for TRPL recycling with the trans-Golgi as a pivotal component for retrograde TRPL transport.

## Data Availability

The original contributions presented in the study are included in the article/Supplementary Material, further inquiries can be directed to the corresponding author.
