# Supplementary figures and images for: tsCRISPR based identification of Rab proteins required for the recycling of Drosophila TRPL ion channel
Source: Front Cell Dev Biol. 2024 Sep 20;12:1444953. doi: 10.3389/fcell.2024.1444953 (PMC11450138; doi:10.3389/fcell.2024.1444953)

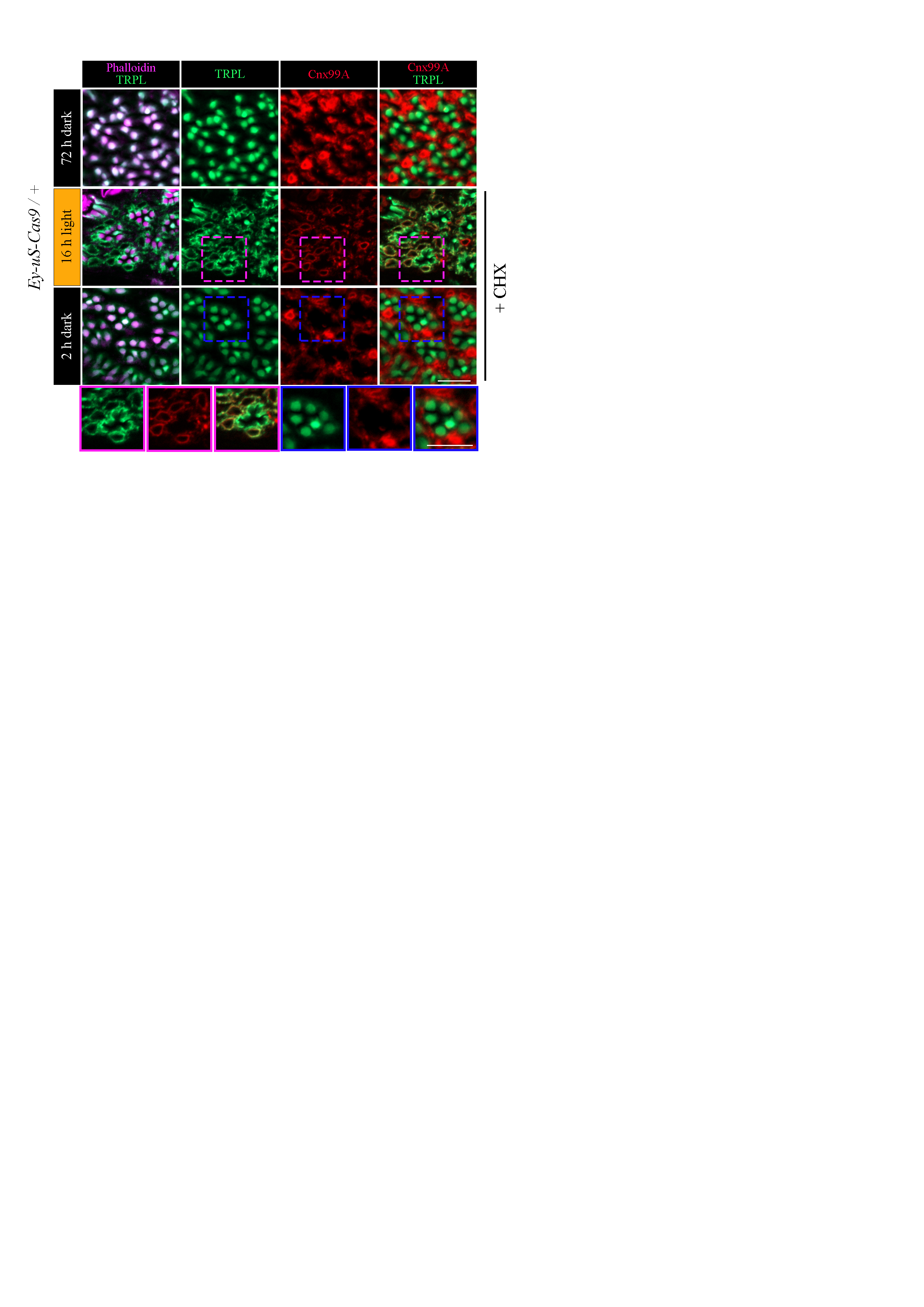

Supplement: Supplementary file 1 [file Image3.TIF]

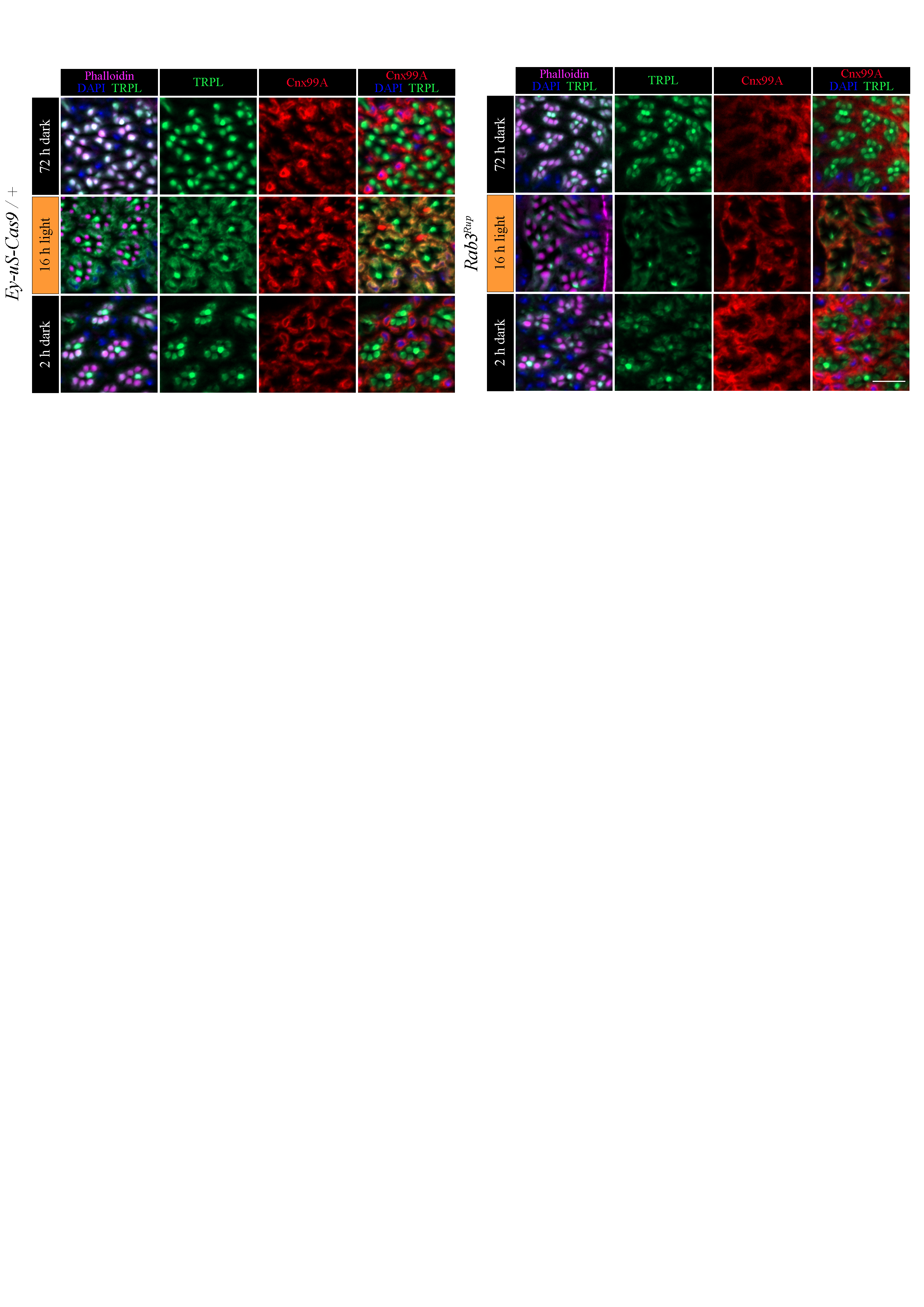

Supplement: Supplementary file 2 [file Image4.TIF]

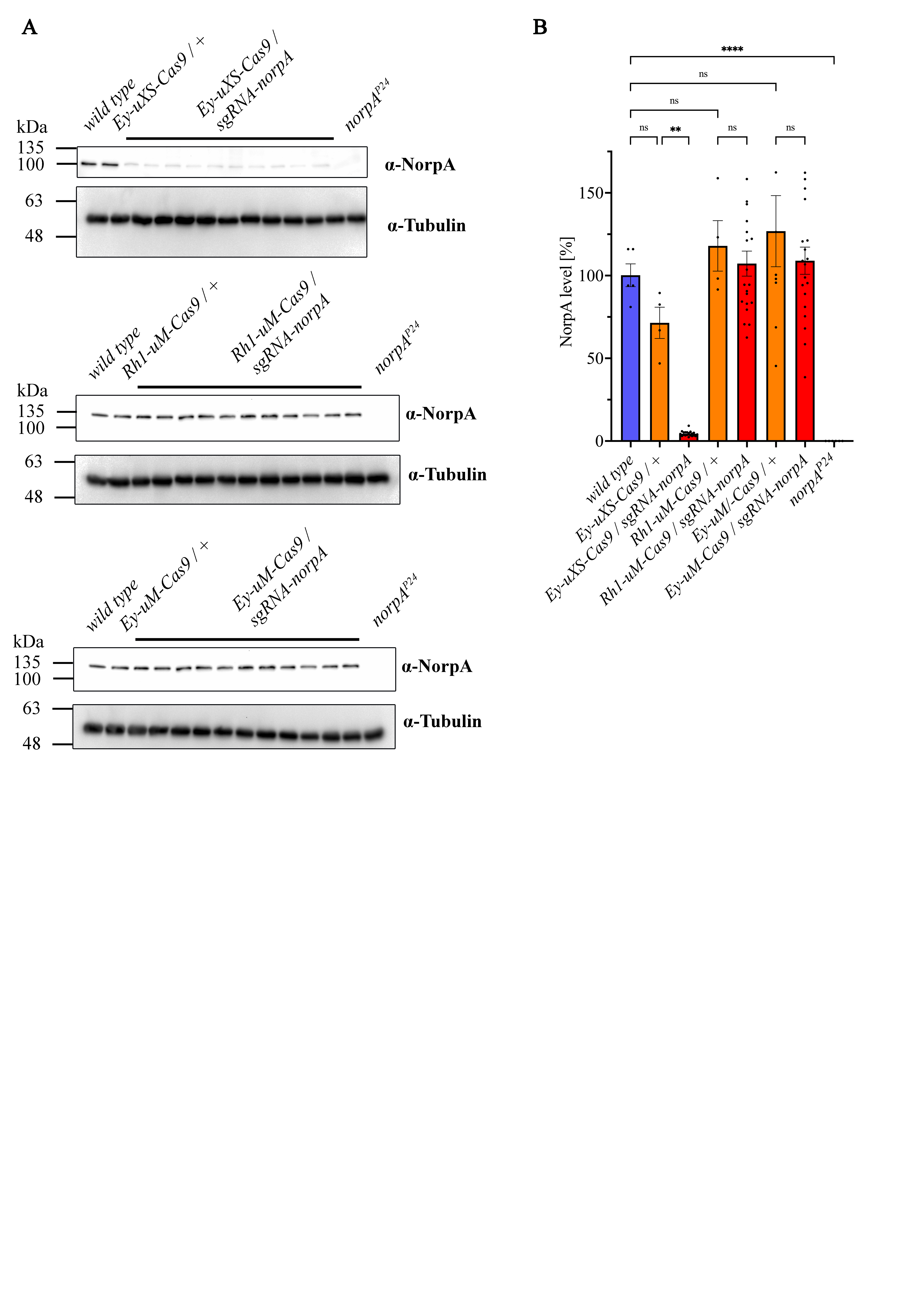

Supplement: Supplementary file 3 [file Image1.TIF]
