# Supplementary material for: tsCRISPR based identification of Rab proteins required for the recycling of Drosophila TRPL ion channel
Source: Front Cell Dev Biol. 2024 Sep 20;12:1444953. doi: 10.3389/fcell.2024.1444953 (PMC11450138; doi:10.3389/fcell.2024.1444953)
Supplement: Supplementary file 4 [file Image2.PDF]

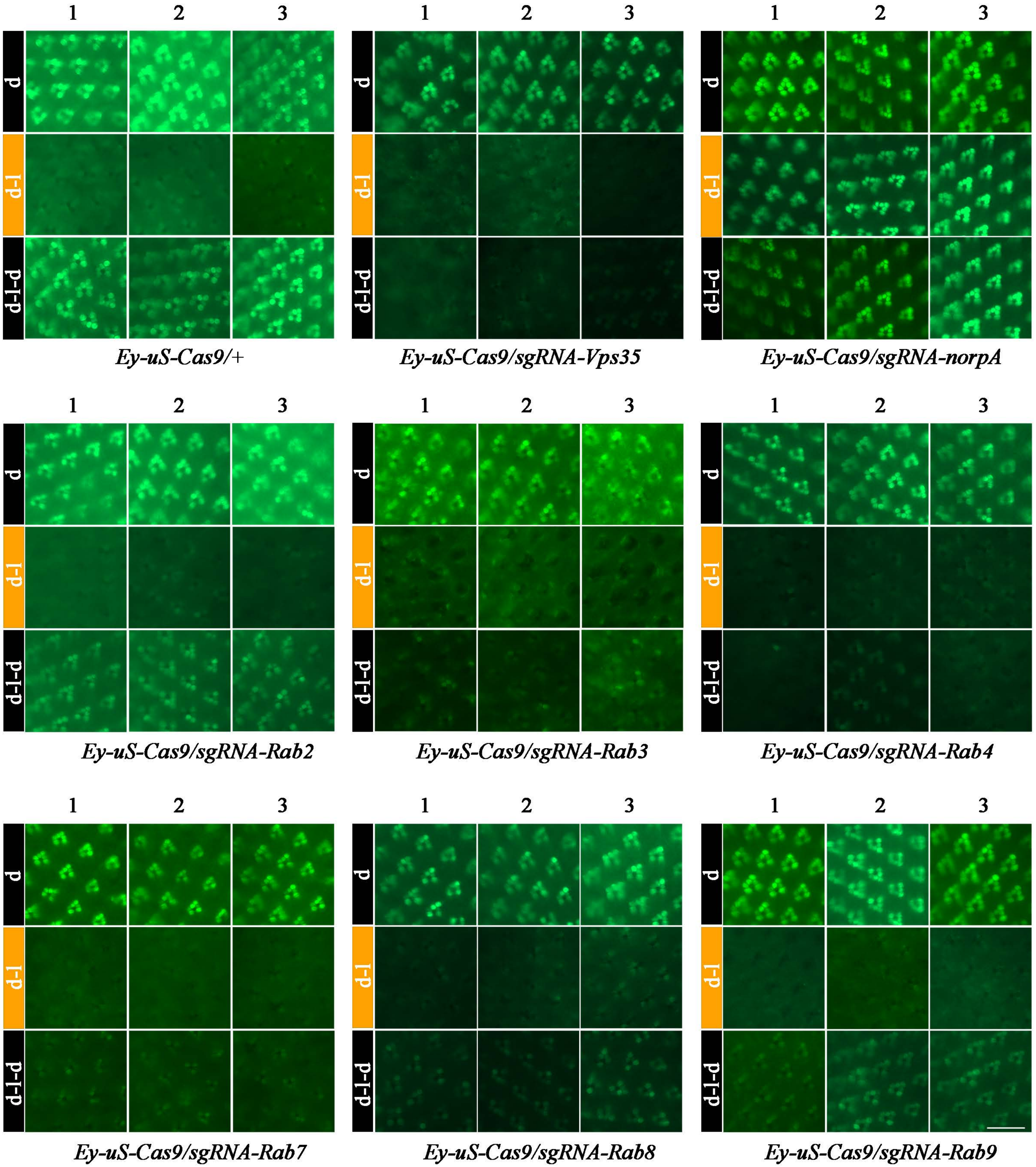
1
2
3
d
d-1
d-1-d
Ey-uS-Cas9/+
Ey-uS-Cas9/sgRNA-Vps35
Ey-uS-Cas9/sgRNA-norpA
Ey-uS-Cas9/sgRNA-Rab2
Ey-uS-Cas9/sgRNA-Rab3
Ey-uS-Cas9/sgRNA-Rab4
Ey-uS-Cas9/sgRNA-Rab7
Ey-uS-Cas9/sgRNA-Rab8
Ey-uS-Cas9/sgRNA-Rab9

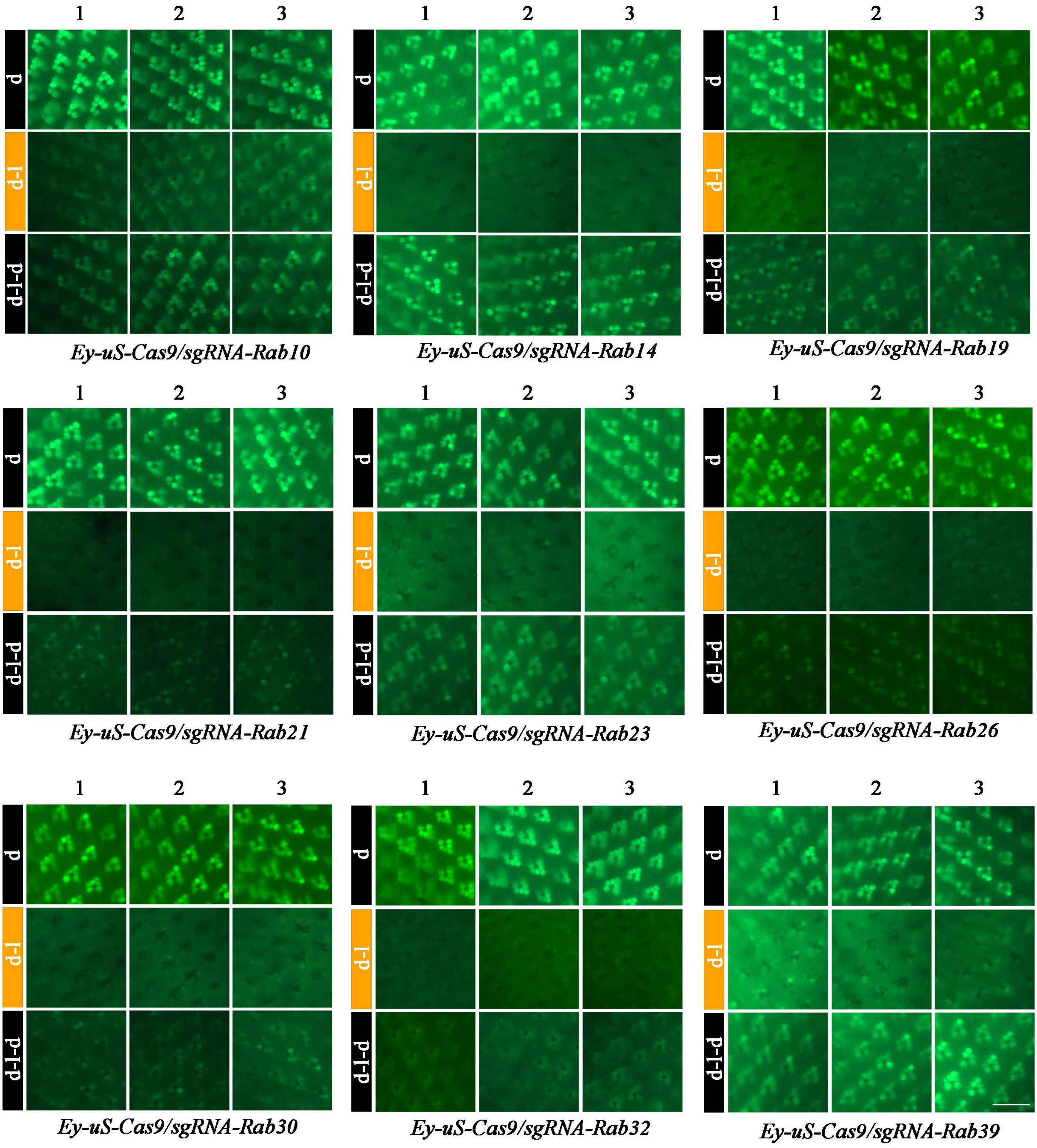
1
2
3
d
d-1
d-1-d
Ey-uS-Cas9/sgRNA-Rab10
Ey-uS-Cas9/sgRNA-Rab14
Ey-uS-Cas9/sgRNA-Rab19
Ey-uS-Cas9/sgRNA-Rab21
Ey-uS-Cas9/sgRNA-Rab23
Ey-uS-Cas9/sgRNA-Rab26
Ey-uS-Cas9/sgRNA-Rab30
Ey-uS-Cas9/sgRNA-Rab32
Ey-uS-Cas9/sgRNA-Rab39

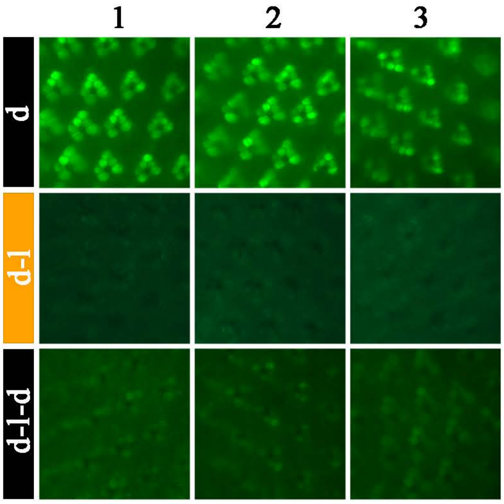

Ey-uS-Cas9/sgRNA-Rab40

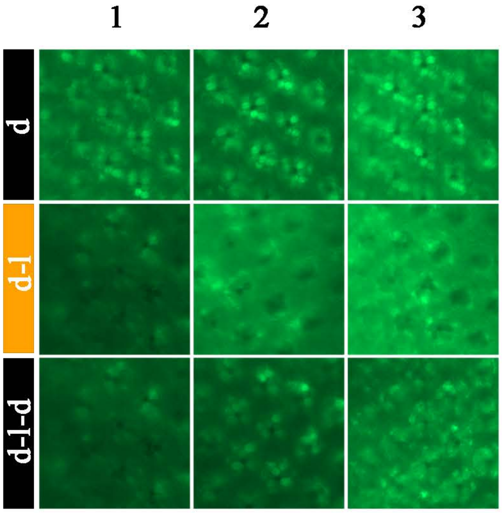

Ey-uS-Cas9/sgRNA-RabX1

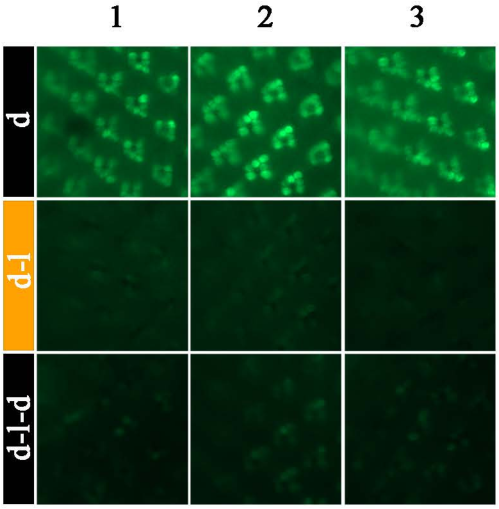

Ey-*uS-Cas9/sgRNA-RabX2*

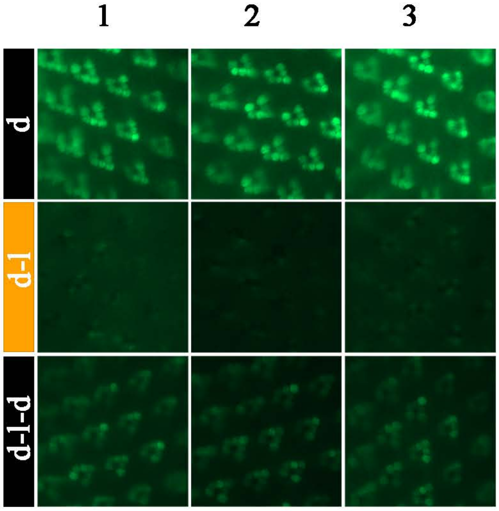

Ey-uS-Cas9/sgRNA-RabX4

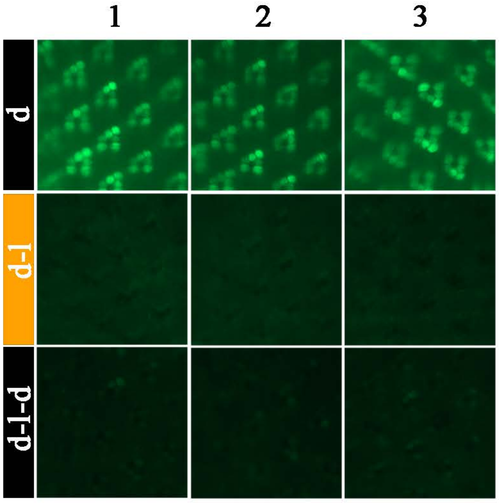

Ey-uS-Cas9/sgRNA-RabX5

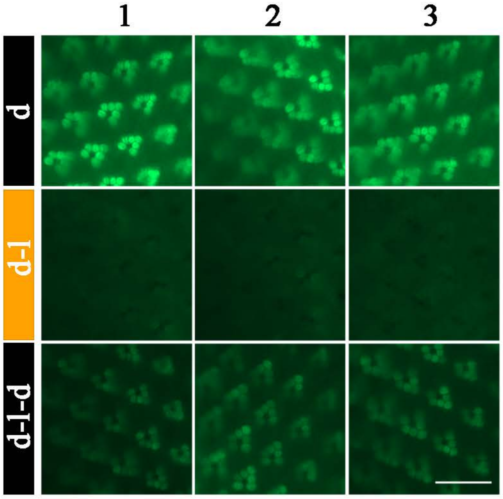

Ey-uS-Cas9/sgRNA-RabX6
