# Supplementary material for: tsCRISPR based identification of Rab proteins required for the recycling of Drosophila TRPL ion channel
Source: Front Cell Dev Biol. 2024 Sep 20;12:1444953. doi: 10.3389/fcell.2024.1444953 (PMC11450138; doi:10.3389/fcell.2024.1444953)
Supplement: Supplementary file 5 [file DataSheet1.DOCX]

**Suppl. Fig. 1: The combination of the *eyeless* promoter or the *Rh1* promoter with *u^M^-Cas9* does not result in an efficient knock-down of NorpA.** (A) Immunoblot analysis assessing NorpA levels in various tissue specific *norpA* knockouts.  Genotypes of the driver constructs containing *Ey* and *Rh1* promoters recombined with different *Cas9* upstream variants are indicated. Single head extracts of 1-2 days old flies were analyzed and NorpA was detected with an α-NorpA antibody. α-Tubulin antibodies served as loading control. All lines were heterozygous for the eye specific promoter and *Cas9*. Molecular weight markers (kDa) are indicated on the left of each blot. (B) Quantification of the immunoblots shown in (A). NorpA signals were normalized to the tubulin signal and the NorpA level of the wild type was set to 100 %. Statistically significant differences were analyzed by a one-way ANOVA test with Bonferroni correction (** p < 0.01, **** p < 0.0001, ns not significant). Error bars: SEM (n = 4-20).

**Suppl. Fig. 2:** **Mutagenesis screen of Rab proteins and their effect on TRPL recycling.** (A) Water immersion micrographs showing TRPL::eGFP localization in *Drosophila* eyes of *Ey-uS-Cas9-TRPL::eGFP/+*, *Ey-uS-Cas9-TRPL::eGFP/sgRNA-norpA* and *Ey-uS-Cas9-TRPL::eGFP/sgRNA-vps35* control flies, as well as the indicated *Rab* CRISPR mutants. Flies were kept in the dark for 72 h after eclosure, kept under orange light for 16 h and were subsequently returned to darkness for another 24 h. For each mutant three individual flies (1-3) are shown. Scale bar 20 µm.

**Suppl. Fig. 3: TRPL is recycled back into the rhabdomeric membrane within 2 hours.**

Localization of TRPL in eye cross-sections of Ey-uS-Cas9 /+ control flies after cycloheximide (CHX) treatment in d, d-l, and d-l-d light conditions. Flies were dark-adapted for 72 h after eclosure, then exposed to orange light for 16 h and were subsequently returned to darkness for another 2 h. CHX was fed to flies when the flies were switched to orange light and was conducted as described previously (Wagner et al., 2022a). Sections were probed with α-Cnx99A and α-TRPL antibodies. Rhabdomeres were stained with phalloidin and nuclei with DAPI. Images at the bottom show magnifications of the indicated areas above. Scale bar: 10 μm.

**Suppl. Fig. 4: *Rab3^Rup^* exhibits a phenotype similar to the observed phenotype in the Rab3 CRISPR mutant.** (A) Localization of TRPL in *Ey-uS-Cas9/+* control flies and *Rab3^Rup^* in d, d-l, and d-l-d conditions. Flies were dark-adapted for 72 h (d), then exposed to orange light for 16 h (d-l), and were subsequently returned to darkness for another 2 h (d-l-d). Cross sections through ommatidia were probed with α-TRPL antibodies and α-Cnx99A antibodies, as indicated. Rhabdomeres were visualized using phalloidin and nuclei were stained with DAPI. Scale bar: 10 μm.
